# Fate of Pup inside the Mycobacterium Proteasome Studied by in-Cell NMR

**DOI:** 10.1371/journal.pone.0074576

**Published:** 2013-09-10

**Authors:** Andres Y. Maldonado, David S. Burz, Sergey Reverdatto, Alexander Shekhtman

**Affiliations:** Department of Chemistry, State University of New York at Albany, Albany, New York, United States of America; The Chinese University of Hong Kong, China

## Abstract

The *Mycobacterium tuberculosis* proteasome is required for maximum virulence and to resist killing by the host immune system. The prokaryotic ubiquitin-like protein, Pup-GGE, targets proteins for proteasome-mediated degradation. We demonstrate that Pup-GGQ, a precursor of Pup-GGE, is not a substrate for proteasomal degradation. Using STINT-NMR, an in-cell NMR technique, we studied the interactions between Pup-GGQ, mycobacterial proteasomal ATPase, Mpa, and *Mtb* proteasome core particle (CP) inside a living cell at amino acid residue resolution. We showed that under in-cell conditions, in the absence of the proteasome CP, Pup-GGQ interacts with Mpa only weakly, primarily through its C-terminal region. When Mpa and non-stoichiometric amounts of proteasome CP are present, both the N-terminal and C-terminal regions of Pup-GGQ bind strongly to Mpa. This suggests a mechanism by which transient binding of Mpa to the proteasome CP controls the fate of Pup.

## Introduction

Inside a cell, macromolecular complexes are assembled along specific pathways necessary to carry out biological functions in the presence of a crowded cytosol [1–3]. Often, during assembly, effector molecules such as ligands or substrates are also present. The presence of these molecules prior to or following the expression of components of the complex can play a regulatory role in the assembly of that complex. Furthermore, the binding of these effector molecules may alter the pathway through which proper, biologically active conformations are achieved. It is not clear *a priori* that the final conformation and commensurate activity of the complex will be different due to this temporal control [4].

One method to study macromolecular complexes inside a cell that affords temporal control over assembly is STINT-NMR [5–7]. STINT-NMR is used to elucidate STructural INTerations between proteins within their native environment by using in-cell NMR [8]. In STINT-NMR, protein over-expression is induced in labeling medium to produce a uniformly labeled [*U*-^15^N] target protein containing NMR-active nuclei; cells are then transferred to non-labeling medium to induce over-expression of the interactor protein. ^15^N- or ^13^C- edited heteronuclear single quantum coherence (^1^H{^15^N}-HSQC or ^1^H{^13^C}-HSQC) NMR experiments are performed to monitor the chemical shifts of target backbone amide or side chain ^13^C-^1^H nuclei as the concentration of interactor is increased. Monitoring the chemical shift changes of the labeled target delineates the intermolecular interaction surface between the target and the interactor(s) [5]. Most importantly, the order of sequential over-expression of target and interactor proteins can be reversed, allowing temporal control over the assembly of the complex.

In this work STINT-NMR was used to examine the interactions between the Ubiquitin-like protein, Pup, in the presence of the Mycobacterium proteasome ATPase, Mpa, and the active 1.2 megadalton proteasome complex, consisting of Mpa and the Mycobacterial proteasome core particle (CP) [9,10]. The importance of this macromolecular complex is underlined by the fact that *Mycobacterium tuberculosis* is particularly resistant to reactive nitrogen intermediates generated by host immune system, and this resistance is related to the proteasome [11] and *mpa* [12]. The *Mtb* proteasome CP consists of 14 copies each of two distinct but related polypeptides, α and β [13]. The overall architecture of the CP is conserved: α- and β-type subunits segregate into four homo-oligomeric 7-member rings (α7β7β7α7) [14]. Two juxtaposed rings of β-type subunits are flanked on top and bottom by a ring of α-type subunits to form the barrel-shaped complex. The main function of the α-rings is to form a gated channel that controls the passage of unfolded substrates into and cleaved particles out of the proteolytic chamber. Studies have shown that *Mtb* Mpa forms 404 kDa hexameric rings similar to AAA+ ATPases found in the eukaryotes [10,12]. Structural analysis predicts that *Mtb* Mpa physically interacts with the α-rings of *Mtb* proteasome CP and plays a role in binding, unfolding and translocating substrates into the proteasome complex [10,15,16].

Proteins that are targeted for degradation in eukaryotes are generally tagged with the Ubiquitin, a small (76 aa) highly conserved regulatory protein [17]. By using *Mtb* Mpa as bait in a bacterial two-hybrid screen of an *Mtb* genomic library searching for potential binding partners of *Mtb* Mpa, the first prokaryotic Ubiquitin-like protein, Pup was discovered [18]. Pup is a 64 amino acid protein that modifies and targets mycobacterium proteins to the proteasome for degradation. Pup is similar in size to Ubiquitin but the two proteins have different sequences and lack structural homology. Pupylated proteins, which have been tagged with Pup, interact with *Mtb* Mpa [15,19].

The *Mtb* proteasome complex presents a tractable in-cell system for studying the interactions between Pup and the proteasomal ATPase, Mpa. Crystal structures of the *Mtb* proteasome CP [14,20], the Pup-Mpa coiled coil domain complex [21], as well as *in vitro* NMR solution studies of Pup-Mpa interactions [19,22,23] are available. *E. coli* is a relevant prokaryotic host that provides a proper milieu for studying the Pup-Mpa interaction without interfering factors. Indeed, *Mtb* Pup ligase, pafA, was overexpressed in *E. coli* to study pupylation of proteins and to prove that no additional factors are required for this process [24]. Individual α- and β-subunits of the *Mtb* proteasome core particle, also called prcA and prcB, respectively, are overexpressed in *E. coli* in an active form, and can self-assemble into the functional *Mtb* proteasome after maturation of β subunit, which proceeds through autocatalytic removal of an N-terminal peptide [13].

Pup contains a di-glycine motif at the penultimate position of the C-terminus, followed by either glutamate (Pup-GGE) or glutamine (Pup-GGQ), depending on the organism [25]. Mass spectrometry revealed that for pupylated substrates in *Mtb*, the C-terminal Gln is not removed, but rather deamidated to Glu prior to being conjugated to substrate lysines [25,26]. Here we present how Pup-GGQ interacts with the Mpa-proteasome complex inside a cell by recreating the final steps in the mycobacterium degradation pathway in *E. coli*. We used Pup-GGQ for our studies since it exists as a free molecule inside the cell, not attached to its target [18].

## Materials and Methods

### Plasmid Construction

#### pTM-Pup

Four separate oligonucleotides, two coding strands (5–1,5–2),, corresponding to amino acids 1-31 and 32-64, respectively, and two complementary strands (3–1,3–2),, were combined to form a complete Pup gene containing flanking 5' *Hind*III and 3' *Bam*HI restriction sites. The oligonucleotide sequences used are (5–1): 5’-CCC A A G C T T ATG GCG CAA GAG CAG ACC AAG CGT GGC GGT GGC GGC GGC GAT GAT GAC GAC ATC GCC GGC AGC ACC GCC GCG GGC CAG GAG CGT CGC GAA AAG CTG ACC GAG GAG ACC GAC-3’ (5–2);: 5’-GAT CTG CTC GAC GAA ATC GAC GAC GTC CTC GAG GAG ACC GCC GAG GAC TTC GTC CGC GCA TAC GTC CAA AAG GGC GGA CAG TGA G G A T C C AA-3’ (3–1);: 5’-GGT CTC CTC GGT CAG CTT TTC GCG ACG CTC CTG GCC CGC GGC GGT GCT GCC GGC GAT GTC GTC ATC ATC GCC GCC GCC ACC GCC ACG CTT GGT CTG CTC TTG CGC CAT A A G C T T GGG-3’ and (3–2);: 5’-TTG G A T C CT CAC TGT CCG CCC TTT TGG ACG TAT GCG CGG ACG AAG TCC TCG GCG TTC TCC TCG AGG ACG TCG TCG ATT TCG TCG AGC AGA TCG TC-3’. Duplexes were formed between (5–1,3–1) and between (5–2,3–2) prior to ligation. The product was restriction digested and ligated into pTM vector to yield pTM-Pup. The resulting plasmid, pTM-Pup, expresses Pup-GGQ from a T7 promoter/lac operator (P_T7_/lacOp), which is induced by isopropyl-β-D-thiogalactoside (IPTG). This plasmid confers kanamycin resistance and contains a pBR322 origin and codes for the *lacI* gene, which encodes for Lac repressor.

#### pASK-Pup

DNA coding for full-length Pup-GGQ was PCR amplified from pTM-Pup using Taq polymerase and the oligonucleotides 5’-AAA AAA G G T C T C TAA TGG CGC AAG AGC AGA CCA AGC GTG G-3’ and 5’-AAA AAA G G T C T C AGC GCT TCA CTG TCC GCC CTT TTG GAC G-3’. The gene was ligated into pASK3+ [IBA] using the *Bsa*I linker sites. The resulting plasmid, pASK-Pup, expresses Pup-GGQ from a tet promoter/operator (P_Tet_/tetOp), which is induced by tetracycline or anhydrotetracycline. This plasmid confers ampicillin resistance and contains an f1 origin and codes for the *tet* gene, which codes for Tet repressor.

#### pASK-PupHis7x

DNA coding for full-length Pup-GGQ was PCR amplified from pASK-Pup by using Taq polymerase and the oligonucleotides 5’-AAA AAA G G T C T C TAA TGG CGC AAG AGC AGA CCA AGC GTG G-3’ and 5’ AAA AAA G G T C T C AGC GCT TCA ATG ATG ATG ATG ATG ATG ATG CTG TCC GCC CTT TTG GAC GTA TGC-3’. The gene was ligated into pASK3+ [IBA] using the *Bsa*I linker sites. The resulting plasmid, pASK-PupHis7x, expresses a C-terminal His-tagged Pup from P_Tet_/tetOp. This plasmid confers ampicillin resistance and contains an f1 origin and codes for the *tet* gene, which codes for Tet repressor.

#### pRSF-Msm Mpa

DNA coding for full-length (aa 1-1149) *Mycobacterium smegmatis* Mpa (*Msm* Mpa) was PCR amplified from isolated genomic DNA extracted from *M. smegmatis* MC^2^ 2700 [27] by using Phusion polymerase and oligonucleotides 5’-AAA AAA G G T A C C ATG GGT GAG TCA GAG CGT TC-3’ and 5’-AAA A A G C T T TCA CAG GTA CTG GCC GAG GTT GG-3’. The gene was ligated into pRSF-1b [Novagen] using the *Kpn*I and *Hind*III linker sites. The resulting plasmid, pRSF-*Msm* Mpa, expresses N-terminal His-tagged *Msm* Mpa from P_T7_/lacOp, which is induced by IPTG. This plasmid confers kanamycin resistance, contains an RSF replication origin and codes for the *lac*I gene, which encodes for Lac repressor.

#### pRSF-Mtb Mpa

DNA coding for full-length (aa 1-1149) *Mycobacterium tuberculosis* Mpa (*Mtb* Mpa) was PCR amplified from isolated genomic DNA extracted from strain H37Rv by using Phusion polymerase and oligonucleotides 5’-AAA AAA G G T A C C ATG GGT GAG TCA GAG CGT TCT GAG G-3’ and 5’-AAA AAG CTT TCA CAG GTA CTG GCC GAG GTT GG3-3’. The gene was ligated into pRSF-1b [Novagen] using the *Kpn*I and *Hind*III linker sites. The resulting plasmid, pRSF-*Mtb* Mpa, expresses N-terminal His-tagged *Mtb* Mpa from P_T7_/lacOp, which is induced by IPTG. This plasmid confers kanamycin resistance, contains an RSF replication origin and codes for the *lac*I gene, which encodes for Lac repressor.

#### pACYCDuet-PrcAB and pACYCDuet-Prc∆AB

Plasmids encoding *Prc*AB and *Prc∆AB* in vector pACYCDuet-1 [13] (a gift from G. Lin, Weil Medical College of Cornell University) were used to overexpress the *Mycobacterium tuberculosis* (*Mtb*) wild type proteasome (pACYCDuet-PrcAB) and the *Mtb* opengate proteasome (pACYCDuet-Prc∆AB). These plasmids express PrcA and His-tagged PrcB, contain a P_T7_/lacOp, a P15A replication origin, confer chloramphenicol resistance and code for the *lac*I gene.

### Sequential over-expression and labeling

*E. coli* strain BL21(DE3) codon+ [Novagen] was co-transformed with pASK-Pup and pRSF-*Msm* Mpa (Pup-GGQ/*Msm* Mpa interaction); or pASK-Pup, pRSF-Mpa and pACYCDuet-PrcAB (Pup-GGQ/*Msm* Mpa/wild type proteasome CP interaction); or pASK-Pup, pRSF-Mpa and pACYCDuet-Prc∆AB (Pup-GGQ/*Msm* Mpa/Opengate proteasome CP interaction) or pASK-PupHis7x, pRSF-Mpa and pACYCDuet-PrcAB (Pup-GGQ-His7x/*Msm* Mpa/wild type proteasome CP interaction); or pASK-PupHis7x, pRSF-Mpa and pACYCDuet-Prc∆AB (Pup-GGQ-His7x/*Msm* Mpa/Opengate proteasome CP interaction). Cells were grown overnight at 37 ^°^C to an OD_600_ of ~1.6 in Luria-Bertani (LB) medium supplemented with 150 mg/L of carbenicillin for cultures containing pASK-Pup or pASK-PupHis7x, and 35 mg/L of kanamycin for cultures containing pRSF-*Msm* Mpa or pRSF-*Mtb* Mpa, and 33 mg/L of chloramphenicol for cultures containing pACYCDuet-PrcAB and pACYCDuet-Prc∆AB. Four protocols were employed, as described below.

#### Protocol 1: Expression of [U-^15^N] Pup-GGQ

Transformed cells from an overnight culture grown in LB containing the appropriate antibiotics were washed once with minimal medium (M9) salts and re-suspended to an OD_600_ of ~0.5 in M9 medium containing the appropriate antibiotics, 0.7 g/L of ^15^N-ammonium chloride as the sole nitrogen source and 0.2% glucose as the sole carbon source. For all induced cultures we substituted ampicillin (100 mg/L) for carbenicillin. The cells were incubated at 37 ^°^C for 10–15 minutes and Pup-GGQ over-expression was induced by adding 2 mg/mL of anhydrotetracycline in dimethylformamide (DMF) to a final concentration of 0.2 µg/mL. Pup-GGQ over-expression was allowed to proceed for up to 4 hours. Following the first induction, a 100 mL sample of culture was collected, the cells were centrifuged, washed twice with 50 mL of 10 mM potassium phosphate buffer [pH 6.5], re-suspended with 1 mL of 10 mM potassium phosphate buffer [pH 6.5] containing 10% glycerol and stored at -80 ^°^C for subsequent NMR analysis. The use of a cryoprotectant is critical to minimize cell lysis due to repeated freeze-thawing. This control sample was used to assess the extent of overexpression and quality of labeling for a given experiment. 50 mL culture samples were collected pre- and post-induction for SDS-PAGE analysis.

#### Expression of Msm Mpa

Following over-expression of labeled Pup-GGQ, the culture was centrifuged and washed once with M9 salts before re-suspending a sufficient number of cells to yield an OD_600_ of ~0.5 in LB medium supplemented with the appropriate antibiotics. The culture was incubated at 37 ^°^C for 10–15 minutes and IPTG was added to a final concentration of 1 mM to induce over-expression of *Msm* Mpa; induction was allowed to proceed for 16 hours. 100 mL samples were collected, centrifuged, washed twice with 10 mM potassium phosphate buffer [pH 6.5], re-suspended with 1 mL of 10 mM potassium phosphate buffer [pH 6.5] containing 10% glycerol and stored at -80 ^°^C for subsequent NMR analysis. 50 mL culture samples were collected pre- and post-induction for SDS-PAGE analysis.

#### Protocol 2: Expression of Msm Mpa

Transformed cells from an overnight culture grown in LB containing the appropriate antibiotics were washed once with LB medium and re-suspended to an OD_600_ of ~0.5 in LB medium containing the appropriate antibiotics. The cells were incubated at 37 ^°^C for 10–15 minutes and IPTG was added to induce over-expression of *Msm* Mpa; induction was allowed to proceed for 16 hours. Following the first induction, a 100 mL control sample of culture was collected, the cells were centrifuged, washed twice with 50 mL of 10 mM potassium phosphate buffer [pH 6.5], re-suspended with 1 mL of 10 mM potassium phosphate buffer [pH 6.5] containing 10% glycerol and stored at -80 ^°^C for subsequent NMR analysis. Samples were collected pre- and post-induction for SDS-PAGE analysis.

Transformed cells from the same overnight culture were washed once with M9 salts and re-suspended to an OD_600_ of ~0.5 in M9 medium containing the appropriate antibiotics, ^15^N-ammonium chloride (0.7 g/L) as the sole nitrogen source and 0.2% glucose as the sole carbon source. The cells were incubated at 37 ^°^C for 10–15 minutes and anhydrotetracycline was added to a final concentration of 0.2 µg/mL to induce over-expression of Pup-GGQ. Induction was allowed to proceed for 4 hours at which time a 100 mL sample of culture was collected and prepared for NMR analysis.

#### Expression of [U-^15^N]-Pup

Following *Msm* Mpa over-expression the culture was washed once with minimal medium (M9) salts and re-suspended to an OD_600_ of ~0.5 in M9 medium containing the appropriate antibiotics, ^15^N-ammonium chloride (0.7 g/L) as the sole nitrogen source and 0.2% glucose as the sole carbon source. The cells were incubated at 37 ^°^C for 10–15 minutes and anhydrotetracycline was added to a final concentration of 0.2 µg/mL to induce over-expression of Pup-GGQ. Induction was allowed to proceed for up to 4 hours. Following the second induction, a 100 mL sample of culture was collected and prepared for NMR analysis. Samples were collected pre- and post-induction for SDS-PAGE analysis.

#### Protocol 3: Expression of [U-^15^N]-Pup-GGQ

Samples were over-expressed, labeled, collected and stored as described in protocol 1.

#### Expression of Msm Mpa and wild type or Opengate proteasome CP

Following Pup-GGQ over-expression and labeling, the culture was centrifuged and washed once with M9 salts before re-suspending a sufficient number of cells to yield an OD_600_ of ~0.5 in LB medium supplemented with the appropriate antibiotics. The culture was incubated at 37 ^°^C for 10–15 minutes and 1.0 M IPTG was added to a final concentration of 1.0 mM to induce over-expression of the *Msm* Mpa/WT proteasome CP complex. Induction was allowed to proceed for 16 hours. 100 mL samples were taken, centrifuged, washed twice with 10 mM potassium phosphate buffer [pH 6.5], re-suspended with 1 mL 10 mM potassium phosphate buffer [pH 6.5] containing 10% glycerol and stored at -80 ^°^C for subsequent NMR analysis. Protocol 3 was also used to co-express *Msm* Mpa and the Opengate proteasome CP. 50 mL Culture samples were taken pre- and post-induction for SDS-PAGE analysis.

#### Protocol 4: Expression of Msm Mpa and wild type or Opengate proteasome CP

Cells from the overnight culture were washed once LB medium and re-suspended to an OD_600_ of ~0.5 in LB medium containing the appropriate antibiotics. For all induced cultures we substituted carbenicillin (150 mg/L) for ampicillin. The cells were incubated at 37 ^°^C for 10–15 minutes and *Msm* Mpa and wild type proteasome over-expression was induced by adding 1.0 M IPTG to a final concentration of 1.0 mM. Induction was allowed to proceed for 16 hours. A 100 mL sample of culture was collected, the cells were centrifuged, washed twice with 50 mL of 10 mM potassium phosphate buffer [pH 6.5], re-suspended with 1 mL 10 mM potassium phosphate buffer [pH 6.5] containing 10% glycerol and stored at -80 ^°^C for subsequent NMR analysis. 50 mL culture samples were taken pre- and post-induction for SDS-PAGE analysis. Protocol 4 was also used to co-express *Msm* Mpa and the Opengate proteasome CP. 50 mL culture samples were taken pre- and post-induction for SDS-PAGE analysis.

Transformed cells from the same overnight culture were washed once with M9 salts and re-suspended to an OD_600_ of ~0.5 in M9 medium containing the appropriate antibiotics, ^15^N-ammonium chloride (0.7 g/L) as the sole nitrogen source and 0.2% glucose as the sole carbon source. The cells were incubated at 37 ^°^C for 10–15 minutes and anhydrotetracycline was added to a final concentration of 0.2 µg/mL to induce over-expression of Pup-GGQ. Induction was allowed to proceed for 4 hours at which time a 100 mL sample of culture was collected and prepared for NMR analysis.

#### Expression of [U-^15^N]-Pup

Samples were over-expressed, labeled, collected and stored as described in protocol 2.

#### Preparation of [U-^13^C, ^15^N] Pup-GGQ

Transformed cells from an overnight culture grown in LB containing the appropriate antibiotics were washed once with minimal medium (M9) salts and re-suspended to an OD_600_ of ~0.5 in M9 medium containing the appropriate antibiotics, 0.7 g/L of ^15^N-ammonium chloride as the sole nitrogen source and 0.2% ^13^C-glucose as the sole carbon source. For all induced cultures we substituted ampicillin (100 mg/L) for carbenicillin. The cells were incubated at 37 ^°^C for 10–15 minutes and Pup-GGQ over-expression was induced by adding 2 mg/mL of anhydrotetracycline in dimethylformamide (DMF) to a final concentration of 0.2 µg/mL. Pup-GGQ over-expression and labeling was allowed to proceed for up to 4 hours. Cells were harvested by centrifugation and stored at -20 °C for analysis.

### SDS-PAGE Analysis

For protocols 1 and 3, gel analyses were performed by taking 100 mL samples at time points 0 h, 2 h, 4 h, and 6 h post Pup-GGQ over-expression followed by 12 h and 24 h post-*Msm* Mpa or *Msm* Mpa/proteasome CP over-expression. For protocols 2 and 4, 100 mL samples were taken at 12 h and 24 h post-*Msm* Mpa or *Msm* Mpa/proteasome CP over-expression followed by 0 h, 2 h, 4 h, and 6 h post Pup-GGQ over-expression. Samples were collected, centrifuged and sonicated. Lysates were analyzed on a 18% acrylamide gel and developed by either Commassie Blue staining or Western blotting using the SuperSignal West HisProbe Kit [Pierce].

### NMR spectroscopy

Cells containing free [*U*-^15^N] Pup-GGQ, [*U*-^15^N] Pup-GGQ/Mpa, or [*U*-^15^N] Pup-GGQ/Mpa/Mtb proteasome CP complexes were re-suspended in 0.5 mL of NMR buffer, 10 mM potassium phosphate, pH 6.5, 90%/10% H_2_O/D_2_O, and transferred to an NMR tube. All NMR experiments were performed at 293K using a Bruker Avance 500 MHz NMR spectrometer equipped with a cryoprobe. We used a Water gate version of the ^1^H{^15^N}-edited HSQC [28]. Data were recorded with 32 transients as 512x64 complex points in proton and nitrogen dimensions, respectively, apodized with a squared cosine-bell window function and zero-filled to 1k{128} points prior to Fourier transformation. The corresponding sweep widths were 12 and 35 ppm in the ^1^H and ^15^N dimensions, respectively. Chemical shifts of [*U*-^15^N] Pup-GGQ inside the cell are slightly different from purified Pup-GGQ. We reassigned the backbone chemical shifts of Pup-GGQ using a clarified lysate of [*U*-^13^C, ^15^N]-Pup-GGQ and a standard suite of triple resonance experiments. To reassign the [*U*-^15^N] Pup-GGQ peaks that changed their positions due to complex formation, we assumed minimum chemical shift changes [29], calculated as Δ_min_ = (δ_H_^2^ + (δ_N_/4)^2^)^^1/2^^, where δ_H_(_N_) represents the change in hydrogen and nitrogen chemical shifts. After each NMR experiment, the cells were pelleted and the ^1^H{^15^N}-HSQC spectrum of the supernatant was collected. No NMR signal was observed above the noise level implying that no leakage or cell lysis was occurring during the experimental acquisition time. Cell viability after in-cell NMR experiments was tested by plating bacteria at 1:10,000, 1:100,000, and 1:1000,000 dilutions on plates containing the appropriate antibiotics before and after in-cell NMR experiments. After counting the colonies, it was established that cell viability was 95 +/- 3%.

The changes in chemical shifts of amide nitrogens and covalently attached amide protons, Δ, were calculated by using Δ = (δ_H_^2^ + (δ_N_/4)^2^)^^1/2^^, where δ_H_(_N_) represents the change in hydrogen and nitrogen chemical shifts. Since the ^1^H{^15^N}-HSQC peaks of side chain amide protons and nitrogens of [*U*-^15^N] Pup-GGQ glutamines do not change positions or broaden during complex formation, we used the intensities of these peaks (I_ref_) to scale the intensities of backbone amide protons and nitrogens. Changes in intensity were calculated by using ΔI = ((I/I_ref_)_free_ -(I/I_ref_)_bound_)/(I/I_ref_)_free_, where (I/I_ref_)_free_ is the scaled intensity of an individual peak in the in-cell spectrum of free Pup-GGQ and (I/I_ref_)_bound_ is the scaled intensity of individual peaks in the in-cell spectrum of the Pup-GGQ/ *Msm* Mpa, Pup-GGQ/ *Msm* Mpa/*Mtb* proteasome CP, or Pup-GGQ/ *Msm* Mpa/*Mtb* Opengate proteasome CP complex. Positive changes in relative intensities denote peak broadening due to binding interactions. Negative changes in intensities are due to overlapping peaks in the bound state. To resolve these overlaps, we assumed that two overlapped peaks are of equal intensity and, consequently, the intensity can be scaled down by two. Due to the qualitative nature of this procedure, all overlapped peaks were marked on the bar diagrams by crosses. To distinguish between residues directly and indirectly affected by complex formation, chemical shift changes above 0.1 ppm and differential peak broadening above 60% were considered to be significant.

### Over-expression and purification of proteins for in vitro assays

*E. coli* strain BL21(DE3) codon+ [Novagen] was individually transformed with pASK-Pup His7x, pRSF-*Msm* Mpa or pRSF-*Mtb* Mpa. Cells expressing Pup-GGQ-His7x were grown in LB medium containing 150 µg/mL of carbenillicin at 37 ^°^C to an OD_600_ of ~0.7 and induced with 2 mg/mL of anhydrotetracycline in DMF to a final concentration of 0.2 µg/mL for 6 h. Cells expressing *Msm* Mpa or *Mtb* Mpa were grown in LB broth containing 35 mg/L of kanamycin at 37 ^°^C to an OD_600_ of ~0.7 and induced with 1.0 mM IPTG for 12 h. The induced cells were harvested by centrifugation at 4000***g*** for 10 min at 4 ^°^C. Cells were suspended in 30 mL of ice-cold lysis buffer, 50 mM NaH_2_PO_4_ [pH 8.0], 300 mM NaCl, 10 mM imidazole, and were lysed by sonication using a Branson sonicator at 300 W for 0.3 s intervals followed by a 1 s rest for a total of 5 min. The lysate was centrifuged at 20000***g*** for 20 min at 4 ^°^C. The supernatant was loaded onto a Ni-NTA resin-filled column (Qiagen). The column was washed with 20 mL of lysis buffer and 30 mL of wash buffer, 50 mM NaH_2_PO_4_ [pH 8.0], 300 mM NaCl, 20 mM imidazole. Fusion proteins were eluted with elution buffer, 50 mM NaH_2_PO_4_ [pH 8.0], 300 mM NaCl, 250 mM imidazole. The eluted proteins were buffer exchanged into 50 mM Tris-HCl [pH 7.5], 150 mM NaCl, 10% (v/v) glycerol, 20 mM MgCl_2_, 1 mM DTT. [*U*-^15^N] Pup-GGQ-His7x was prepared in the same way except that LB broth was replaced by M9 medium containing 0.7 g/L of ^^15^^NH_4_Cl as the sole nitrogen source. SDS-PAGE analysis revealed bands at 15 kDa for Pup, 65 kDa for *Msm* Mpa and 66 kDa for *Mtb* Mpa. Protein concentrations were calculated by using the Bradford method.

*E. coli* strain C41 DE3+ [Novagen] was individually transformed with pACYCDuet-Prc∆AB or pACYCDuet–PrcAB. Single colonies were used to inoculate the Overnight Express Autoinduction System 1 medium (Novagen) and grown at 37 ^°^C for 18 h. Cells were centrifuged at 6000***g*** for 20 min, re-suspended in ice-cold lysis buffer and lysed using a Branson sonicator at 300 W, for 0.3 s pulse intervals followed by a 1 s rest for a total of 5 min. The remaining purification steps were carried out at 4 °C. Supernatants were centrifuged at 20000***g*** for 30 min, mixed with Ni^2+^-nitrilotriacetate acid (Ni-NTA) agarose beads and incubated for 2 h or overnight so that the assembled proteasome CP (PrcA and PrcB) containing His-tagged PrcB binds to the NiNTA matrix. The beads were washed four times with wash buffer and the proteasome CP complex eluted using elution buffer. Fractions containing PrcB and PrcA, as judged by Coomassie staining of SDS-PAGE gels, were pooled and purified on Hi-trap Q-Sepharose (Amersham) by using FPLC (Amersham) with a gradient of buffer in 50 mM Tris-HCl, pH 7.5, 0–1 M NaCl. The eluted proteins were buffer exchanged into 100 mL of reaction buffer R.

### In vitro degradation assay

*In vitro* degradation of Pup-GGQ by the Mpa/proteasome CP complex was performed as described by Striebel et al. (2010) ( [15]). In summary, the reaction was carried out in buffer R containing 25 mM phosphocreatine (Sigma), 1 U/mL creatine phosphokinase (Sigma), 0.3 µM *Msm* Mpa, 0.4 µM Opengate proteasome CP, 3 µM Pup-GGQ and 5 mM ATP. Assays in which WT proteasome CP was used in place of the Opengate proteasome CP or where *Mtb* Mpa was used in place of *Msm* Mpa were carried out under identical conditions. Reactions were performed at 25 ^°^C. Samples were collected at 0, 30, 60, 120, 240 min and quenched by adding SDS-sample buffer and analyzed by SDS–PAGE and/or Western blots using the SuperSignal West HisProbe Kit [Pierce].

### Proteasome activity assay

The WT and Opengate proteasome CPs were purified as described and exchanged into buffer PA, 20 mM Hepes [pH 7.5], 0.5 mM EDTA. Proteasome CPs (10 ng of each subunit) were incubated at 37 ^°^C with 20 µM Suc-LLVY-AMC in a 96-well black plate (Corning) inside a fluorescence spectrophotometer with continuous stirring. The release of AMC was monitored at λ_em_ =440 nm using λ_ex_ = 360 nm; experiments were performed in triplicate. Inhibition of proteasome CPs was accomplished by adding (0.8 nmol) of Bortezomib to each reaction to ensure that degradation occurred via the proteasome.

## Results

### Pup-GGQ is disordered inside E. coli

**Figure 1:**
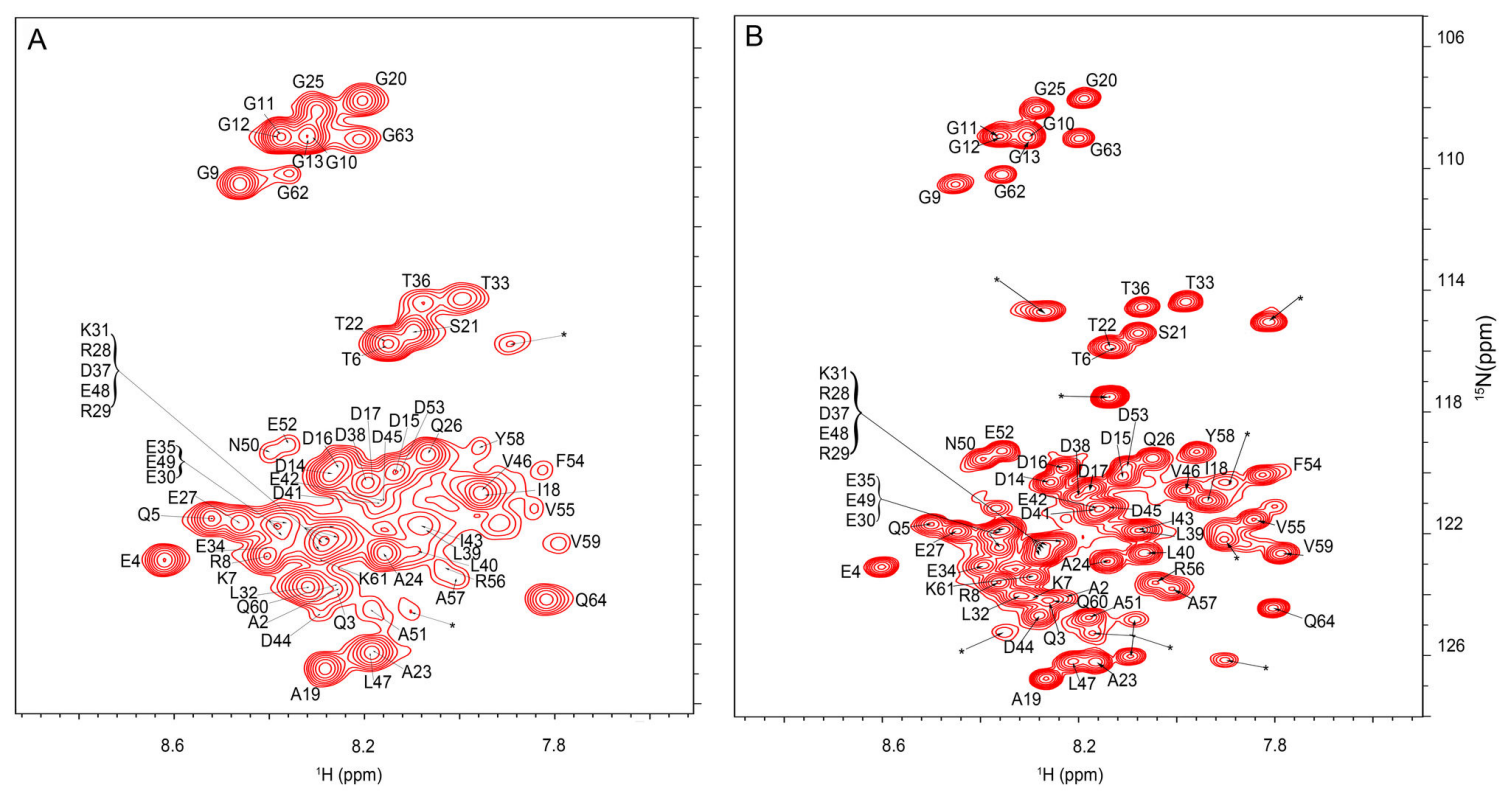
Pup is disordered inside *E. coli*. A. In-cell ^1^H{^15^N}-HSQC spectrum of [*U*-^15^N] Pup-GGQ. B. *In vitro* ^1^H{^15^N}-HSQC spectrum of [*U*-^15^N] Pup-GGQ. The comparatively high viscosity of the cytosol, relative to that of the cell lysate, results in a lower rate of tumbling and consequent peak broadening in the in-cell spectrum. Conversely, the lower viscosity of the cell lysate allows faster tumbling and generates sharper resonances in the *in vitro* spectrum. For quantitation of changes in chemical shifts and peak intensities see Figure **S1**. Asterisks denote NMR peaks originating from small metabolites.

Pup is a small intrinsically unstructured protein with NMR relaxation properties favorable for in-cell NMR analysis [22,23,30,31]. To determine if intracellular Pup contains any regions of induced secondary structure due to macromolecular crowding, [*U-* ^15^N] Pup-GGQ was over-expressed in *E. coli* and an in-cell ^1^H{^15^N}-HSQC NMR spectrum was collected (Figure **1A**). The spectrum of Pup exhibited a signal to noise ratio of better than 5:1 and contained well-resolved backbone amide peaks. To determine that the signals were due solely to intracellular protein, after collecting the spectrum, the sample was centrifuged and the supernatant was examined. No NMR signal was observed above the noise level implying that no leakage or cell lysis was occurring during the experimental acquisition time (Figure **S1A**).

After obtaining the in-cell spectrum, cells containing [*U-* ^15^N] Pup-GGQ were lysed and the spectrum of the lysate was collected (Figure **1B**). The peaks from the *in vitro* spectrum are sharpened, relative to those of the in-cell spectrum, because of the increased rate of tumbling associated with the decreased viscosity of the lysate. The chemical shift of all but three peaks, D16, V46, and D53, from both the in-cell and lysate spectra are found to be within 0.1 ppm of each other (Figures **S1B** and **S1C**). Changes in protein conformation typically result in significant/larger changes (>0.5 ppm) in the chemical shifts. Since only minor changes in chemical shifts and peak intensities were observed, we conclude that Pup remains an intrinsically disordered protein *in vivo*.

### Both the N- and C-termini of Pup engage the proteasome ATPase, Mpa

Pup residues 21 through 51 exhibit a propensity to form a transient α-helical structure [22,23]: ^13^C chemical shifts of α- and β-carbons in this region of the protein are consistent with partial ordering of the structure [32], while the lack of dispersion in the amide region of the NMR spectrum suggests a disordered state. The crystal structure of the Pup-Mpa complex shows a helix conformation when Pup-GGE binds to the coiled coil domain of Mpa [21]. The binding induces a stable helical conformation encompassing amino acids 21-51 of Pup-, while the N- (aa 1-21) and C-termini (aa 52-64) remain unstructured in the Pup-Mpa complex [21]. To investigate the structural role of the interaction between the α-helix and Mpa under in-cell conditions, STINT-NMR [6] was used to characterize the interaction surface of Pup-GGQ when bound to *Mycobacterium smegmatis* Mpa, *Msm* Mpa.

**Figure 2:**
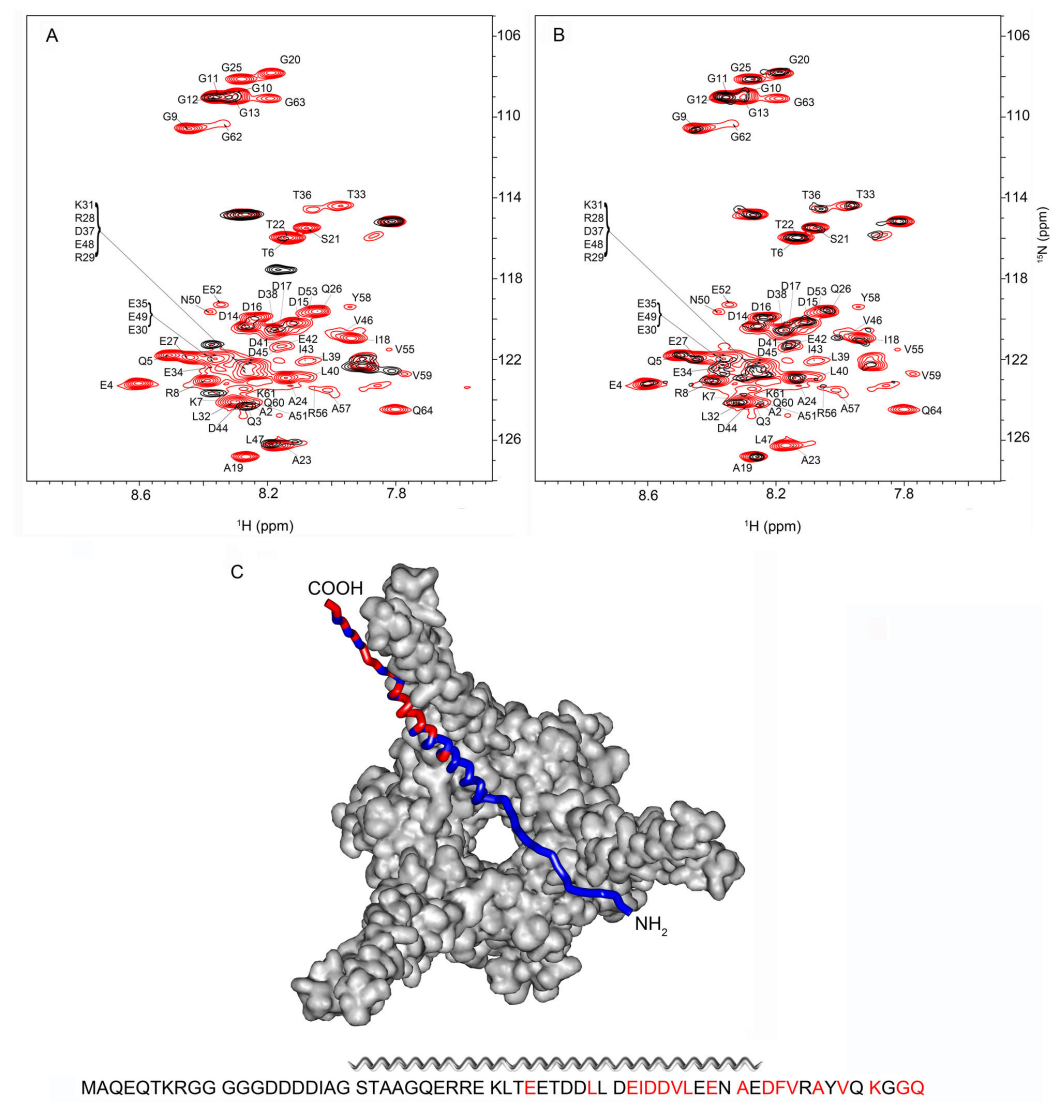
Pup-GGQ interacts with *Msm* Mpa through its N-terminus, C-terminus and α-helix. A. Overlay of *E. coli* ^1^H{^15^N}-HSQC spectra showing [*U-* ^15^N] Pup-GGQ after 4 h of over-expression (red), and after 4 h of over-expression following 16 h of *Msm* Mpa over-expression (black). B. Overlay of *E. coli* ^1^H{^15^N}-HSQC spectra showing [*U-* ^15^N] Pup-GGQ after 4 h of over-expression (red) followed by 16 h of *Msm* Mpa over-expression (black). C. Ribbon model of Pup docked with *Msm* Mpa. The sequence of Pup-GGQ is below the image. Residues that broadened the most upon complex formation with *Msm* Mpa are shown in red. The model is created by Swiss-PDB Viewer [43] based on the Pup-GGE-*Mtb* Mpa complex [21]. For quantitation of changes in chemical shifts and peak intensities see Figure **S2**.

Mpa was first overexpressed in non-labeling medium followed by over-expression of Pup-GGQ in [*U-* ^15^N] labeling medium. In-cell ^1^H{^15^N}-HSQC NMR spectra were collected as the concentration of Pup-GGQ increased (Figure **2A**). Because Mpa is in excess and the binding affinity of Pup for full length Mpa is sub-micromolar [21], as Pup-GGQ is over-expressed, a complex is expected to form. Depending on the chemical exchange rate between the free and bound states of the target, affected NMR peaks, corresponding to backbone amides, can shift, broaden their line shape or disappear completely. In the case of peak broadening, changes are monitored relative to the spectrum of the free target since only this species gives rise to visible peaks.

Indeed, peak broadening was observed when Pup-GGQ was over-expressed in the presence of Mpa. The most pronounced effects were seen for residues K7 in the N-terminal region, L39, E42, I43, D44, D45, L47, E49, and A51 of the α-helix region, and D53, F54, V55, A57, V59, K61, G63 and Q64 in the C-terminus of the protein (Figure **2A**, **S2A, and S2B**). The largest changes in chemical shifts are primarily localized to the C-terminal half of the α-helix (Figure **S2A**). These results suggest that several regions of Pup-GGQ, including most of the C-terminal region (residues 52-64), part of the α-helix (residues 39-51), as well as a portion of the N-terminal region of Pup-GGQ are involved in the interaction with *Msm* Mpa (Figure **2C**). The engagement of N-terminal residues of Pup by Mpa is a novel finding that likely results from interactions that can only be observed *in vivo* or in-cell.

To deduce whether the presence of Pup-GGQ affects the assembly of *Msm* Mpa and therefore the formation of the Pup-GGQ/ Mpa complex, Pup-GGQ was first over-expressed in [*U-* ^15^N] labeling medium followed by over-expression of Mpa in non-labeling medium. In-cell ^1^H{^15^N}-HSQC NMR spectra were collected as the concentration of Mpa increased (Figure **2B**). The differential broadening observed in the resulting spectra are characteristic of intermediate exchange and reflect an equilibrium between free and bound Pup-GGQ since Pup-GGQ is in excess and Mpa is not over-expressed to a sufficiently high level to form a large population of a complex. In general, the same regions of Pup-GGQ are most strongly perturbed as in the previous experiment, and while the magnitudes of the chemical shift changes are comparable (Figure **S2C**), the magnitudes of the intensity changes are reduced, consistent with sub-stoichiometric populations of Pup-GGQ and Mpa (Figure **S2D**). The order of expression appears to have no significant effect on the regions of Pup-GGQ affected by Mpa binding. We conclude that *Msm* Mpa assembles into the same conformational state regardless of the absence or presence of a high concentration of its physiological ligand.

### Pup-GGQ is not degraded when bound to Mpa-proteasome complex in-cell

To examine the interaction of Pup-GGQ with Mpa in the context of the intact proteasome, STINT-NMR experiments were performed in which Pup-GGQ and the interactor complex, consisting of Mpa and the *Mtb* wild type (WT) proteasome CP were over-expressed. Simultaneous over-expression of Mpa and the *Mtb* WT proteasome CP allows the assembly of a functional mycobacterial proteasome complex in *E. coli*. However, high levels of over-expression of *Mtb* Mpa and the *Mtb* proteasome CP could not be achieved in the same cell. Because of 91% sequence identity between *Msm* Mpa and *Mtb* Mpa, and the ability to form the same hexameric structure, *Msm* Mpa is often used in functional studies of *Mtb* proteasome activity [25,33]. To ensure the highest levels of protein over-expression, we used *Msm* Mpa in all experiments.

**Figure 3:**
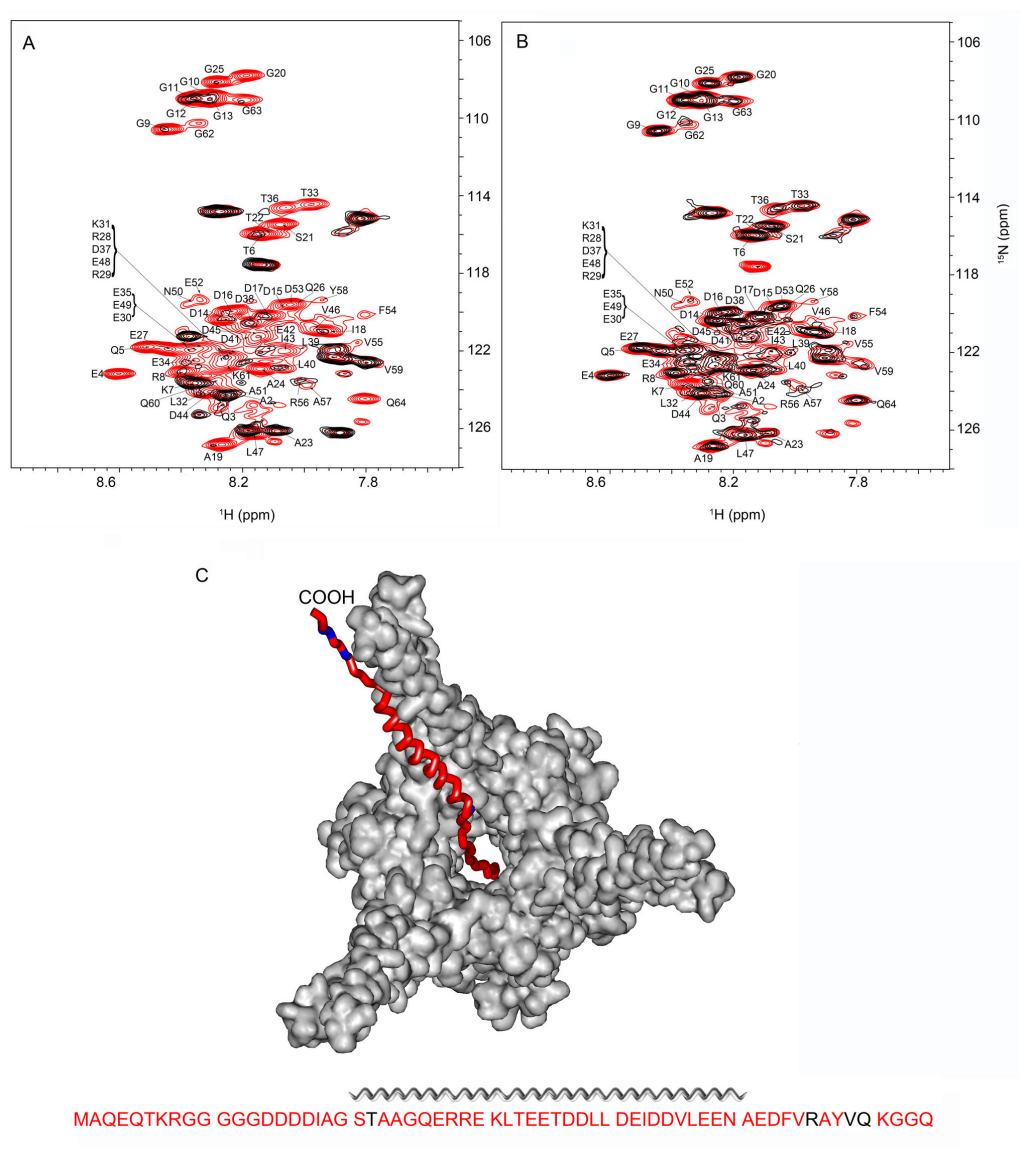
Pup-GGQ is extensively engaged with *Msm* Mpa in the wild type proteasome complex. A. Overlay of *E. coli* ^1^H{^15^N}-HSQC spectra showing [*U-* ^15^N] Pup-GGQ after 4 h of over-expression (red), and after 4 h of over-expression following 16 h of *Msm* Mpa/WT proteasome complex over-expression (black). B. Overlay of *E. coli* ^1^H{^15^N}-HSQC spectra showing [*U-* ^15^N] Pup-GGQ after 4 h of over-expression (red) followed by 16 h of *Msm* Mpa/WT proteasome complex over-expression (black). C. Ribbon model of Pup docked with *Msm* Mpa in the context of the *Msm* Mpa/WT proteasome complex. The sequence of Pup-GGQ is below the image. Residues that broadened the most upon complex formation with the proteasome complex are shown in red. The model is created by Swiss-PDB Viewer [43] based on the Pup-GGE-*Mtb* Mpa complex [21]. For quantitation of changes in chemical shifts and peak intensities see Figure **S3**.

To create the Pup-GGQ/proteasome complex, simultaneous over-expression of *Msm* Mpa and the α and β subunits of the *Mtb* proteasome CP were induced off compatible plasmids in non-labeling medium followed by over-expression of [*U*-^15^N]-Pup-GGQ in labeling medium. In-cell ^1^H{^15^N}-HSQC NMR spectra were collected as the concentration of Pup-GGQ increased (Figure **3A**). Under these conditions, a complex between Pup-GGQ and the *Msm* Mpa/*Mtb* proteasome CP is formed. Signal intensities are uniformly broadened for all but T22, R56, V59, and Q60 of Pup-GGQ reflecting the interaction of Pup-GGQ with the much larger proteasome complex (Figure **S3**). As in the interaction of Pup-GGQ with *Msm* Mpa, the largest changes in chemical shifts are primarily localized to the C-terminal region of the α-helix (Figures **3A** and **S3A**). Thus, Pup-GGQ appears to be more extensively engaged with *Msm* Mpa in the *Msm* Mpa/*Mtb* proteasome CP complex than with *Msm* Mpa alone, and involves residues from the N-terminus, α-helix and C-terminus of the protein (Figure **3C**). We believe that the complete disappearance of the NMR spectrum is due to the N-terminal tail of Pup falling into the central cavity of the Mpa hexamer.

The order of over-expression was reversed, i.e. [*U*-^15^N] Pup-GGQ followed by *Msm* Mpa and the *Mtb* proteasome CP, and in-cell ^1^H{^15^N}-HSQC spectra were acquired. As observed for the Pup-GGQ/*Msm* Mpa interaction, the resulting spectra are characteristic of intermediate/slow exchange and the inability to form a large population of complex implying that the end point to the titration was not reached (Figure **3B**). A smaller set of residues are broadened, L32, L40, and E42, in the α-helix (Figure **3B and S3**). As seen with previous interactions, the largest chemical shifts affect primarily residues in the C-terminus of the α-helix and the C-terminal region of the protein (Figure **S3C**).

Pup is conjugated to proteins targeted for degradation through its C-terminus; an interaction confirmed by examining N-terminal deletion mutants of Pup [15,34]. Furthermore, the N-terminus is necessary for pupylated substrates to be directed into the Mpa central pore [15]. It was expected that in the presence of the *Msm* Mpa/*Mtb* proteasome CP complex, the N-terminus of Pup-GGQ would be engaged, thereby initiating degradation by the proteasome. As the concentration of the *Msm* Mpa/*Mtb* proteasome CP complex increased, no degradation of Pup-GGQ was observed, as monitored by Western blots and/or in-cell NMR. We rationalized that the *Msm* Mpa/*Mtb* proteasome CP complex may require additional factors not present in *E. coli* to remove inhibition for proteolysis of Pup-GGQ.

**Figure 4:**
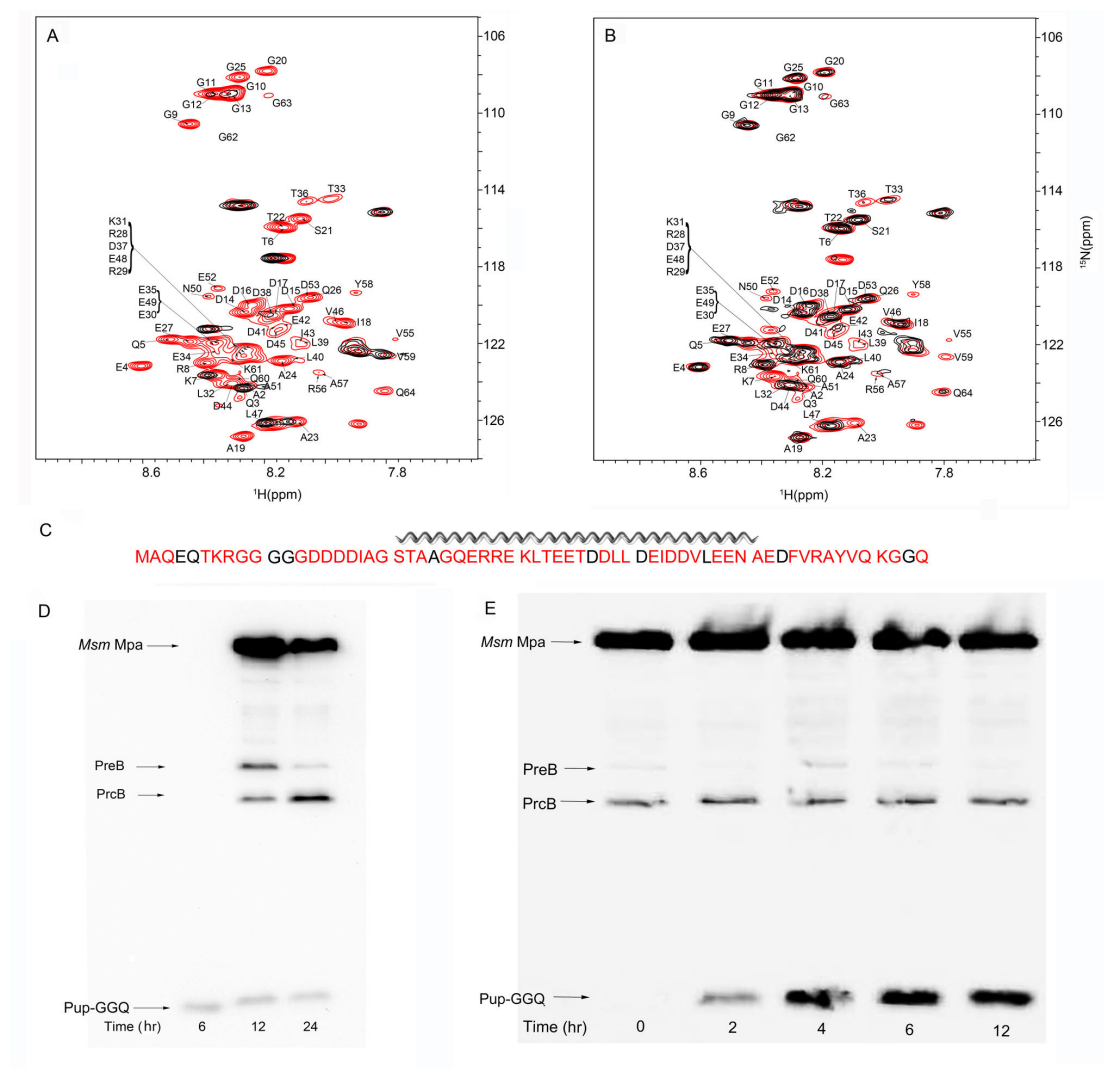
The *Msm* Mpa/Opengate proteasome complex does not degrade Pup-GGQ. A. Overlay of *E. coli* ^1^H{^15^N}-HSQC spectra showing [*U-* ^15^N] Pup-GGQ after 4 h of over-expression (red), and after 4 h of over-expression following 16 h of *Msm* Mpa/Opengate proteasome complex over-expression (black). B. Overlay of *E. coli* ^1^H{^15^N}-HSQC spectra of [*U-* ^15^N] Pup-GGQ after 4 h of over-expression (red) followed by 16 h of *Msm* Mpa/Opengate proteasome complex over-expression. C. Sequence of Pup-GGQ showing the greatest intensity changes upon complex formation in red. D & E. Western blots showing the effect of *Msm* Mpa and the Opengate proteasome CP over-expression on Pup-GGQ induction, probed with anti-His-tag HRP-conjugated antibody. D. Pup-GGQ was over-expressed first for 6 h, followed by over-expression of *Msm* Mpa/Opengate proteasome complex for up to 24 h. PrcB is the proteasomal β-subunit, PreB is unprocessed proteasomal β-subunit. E. Mpa-Proteasome complex was over-expressed for 16 h, followed by over-expression of Pup-GGQ for up to 12 h. Over-expression of the proteasome complex does not result in the degradation of Pup-GGQ. For quantitation of changes in chemical shifts and peak intensities see Figure **S4**.

We substituted the Opengate CP for the WT proteasome CP to circumvent this requirement [12,15]. The Opengate CP lacks the eight N-terminal amino acids of the α-chains that regulate gated entry into the proteasome complex, and shows increased proteolytic activity for its substrates [14,15]. STINT-NMR experiments were carried out as described above to form the *Msm* Mpa/Opengate proteasome complex and in-cell ^1^H{^15^N}-HSQC NMR spectra were collected. As the concentration of [*U*-^15^N]-Pup-GGQ increased in the presence of the *Msm* Mpa/Opengate proteasome, a complex is formed (Figure **4**). The signal intensities observed are uniformly broadened for the majority of Pup-GGQ residues, albeit to a lesser extent than seen with the *Mtb* proteasome CP, implying that most of the protein is still engaged with the proteasome complex (Figure **4A, 4C** **, and S4**). A small number of Pup-GGQ residues exhibit significant changes in chemical shifts, D38, V46 and N50 of the α-helix (Figure **S4A**). The interaction between Pup-GGQ and the *Msm* Mpa/Opengate proteasome complex is very similar to but less extensive than that of Pup-GGQ and the *Msm* Mpa/*Mtb* proteasome CP complex, affecting primarily the C-terminus of the α-helix, the C-terminal region and parts of the N-terminus of Pup-GGQ. These differences suggest that the Pup-GGQ-Mpa complex may contact the 8-amino acid terminal tail of the WT α-subunit when bound to the proteasome CP.

As the concentration of *Msm* Mpa/Opengate proteasome complex was increased in the presence of [*U*-^15^N]-Pup the resulting in-cell ^1^H{^15^N}-HSQC NMR spectra show that the most pronounced chemical shift changes and intensity changes are limited to the C-terminal half of theα-helix and the C-terminal region of Pup-GGQ (Figure **4B and S4**). As before, the relatively small number of perturbed residues is due to the fact that the titration endpoint was not reached and the resulting spectra represent an equilibrium between free and bound Pup-GGQ. Western blots indicate that the β subunit of the CP is processed and there is no degradation of Pup-GGQ resulting from its interaction with the proteasome complex (Figure **4D and 4E**). The STINT-NMR results coupled with Western blots provide compelling evidence that the N-terminus of Pup is engaged by the *Msm* Mpa/proteasome complex, but is not degraded.

Pup-GGE has been shown to be a poor substrate for proteasomal degradation [15]. We used the in-cell *E. coli* system to determine whether or not Pup-GGE is degraded under these conditions. Pup-GGE cloned into the pASK-3+ expression vector failed to produce a visible band on Western blots. However, Pup-GGE cloned into the pCDF-1b expression vector, which utilizes the strong T7 phage promoter for protein over-expression, produced a weak band on Western blots (Figure **S5**). Over-expression of Pup-GGE was suppressed following simultaneous over-expression of *Msm* Mpa and the *Mtb* proteasome CP (Figure **S5**). The over-expression peaked at 12 h and decreased over time suggesting that Pup-GGE may be degraded by an active proteasome in *E. coli*, is subject to proteolysis by endogenous *E. coli* proteases, or may be a result of cell death. Proteasomal degradation of Pup-GGE [15] and pupylated substrates [15,34] was reconstituted *in vitro*. In both cases, degradation was extremely slow, requiring more than 6 hours to detect. Our results are consistent with the conclusion that Pup-GGE is a very poor substrate for proteasomal degradation both in-cell and *in vitro.*

### Re-creation of the Pup proteasome pathway in vitro reveals that Pup-GGQ is not degraded

**Figure 5:**
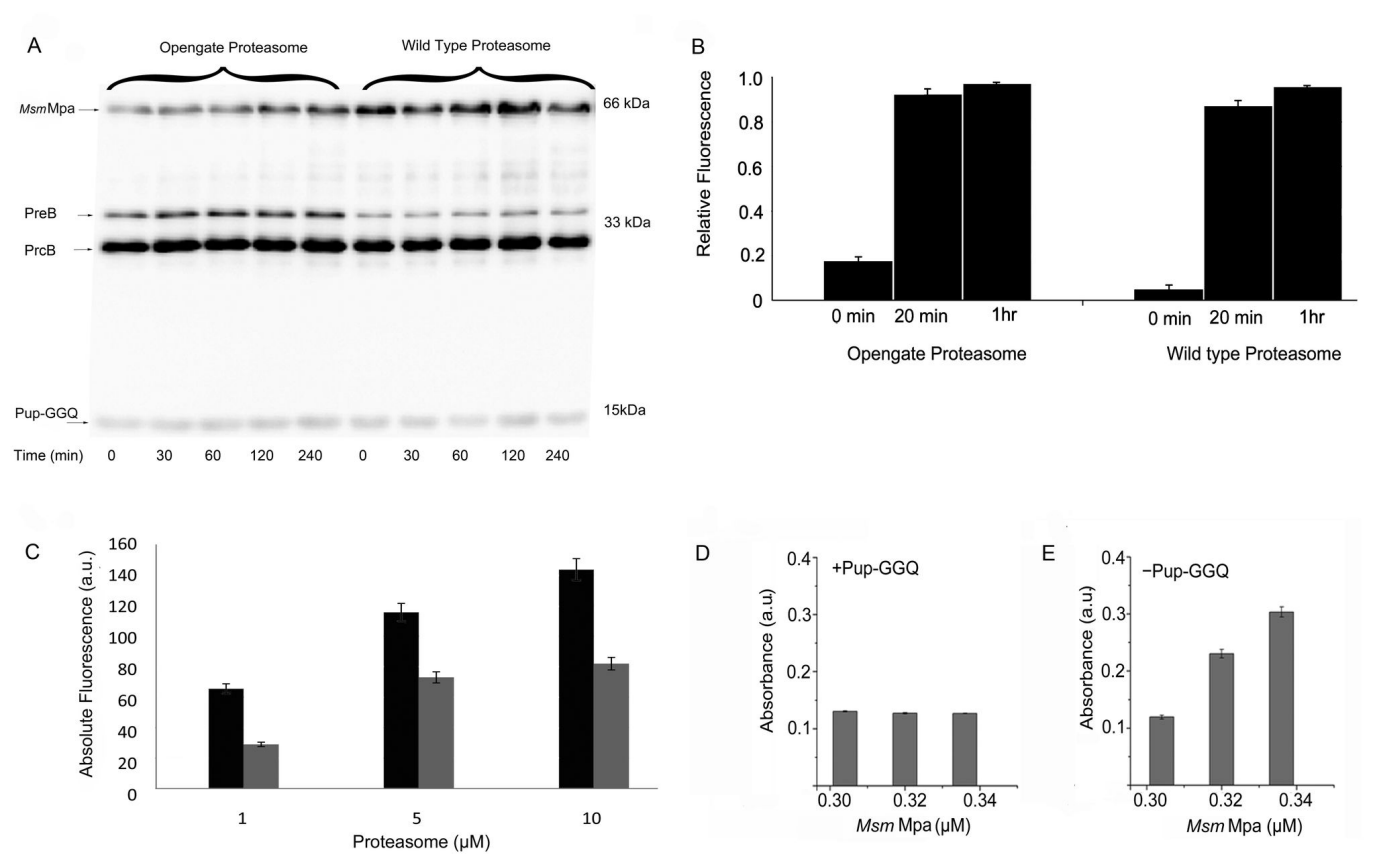
Pup-GGQ is not degraded by the *Mtb* proteasome *in vitro*. A. Western analysis of the Pup-GGQ degradation assay: (Lanes 1-5) 0.1 µM *Msm* Mpa, 0.3 µM Opengate proteasome CP and 15 µM Pup-GGQ; (Lanes 6-10) 0.15 µM *Msm* Mpa, 0.2 µM WT proteasome CP and 15 µM Pup-GGQ. B. Activity assay for the Opengate and WT proteasome CPs. Both WT and Opengate proteasome CPs exhibit increased fluorescence after 1 h due to cleavage of the Suc-LLVT-AMC substrate indicating proteasome activity. C. Inhibition of WT proteasome CP activity by Bortezomib. Proteasome activity in the absence (black) and presence (gray) of Bortezomib. All experiments were performed in triplicate. The data are presented as mean +/- SEM. See also Figure **S6**.

Proteins that are targeted for degradation in eukaryotes are tagged with Ubiquitin, a small (76 aa), highly conserved regulatory protein [17]. One of the key motifs of this pathway is the highly conserved mechanism of how Ubiquitin is recycled. Although Pup may be recycled [40], it has also been reported to be degraded when bound to target proteins and when free in the cytosol [15]. To determine whether Pup-GGQ is degraded by either the *Msm* Mpa/WT proteasome or *Msm* Mpa/Opengate proteasome complex, the degradation assay of Striebel et al. [15] was employed. The reaction was initiated by adding ATP to a final concentration of 5 mM, samples were collected at 30 minute intervals, quenched with loading buffer and analyzed by SDS-PAGE. No degradation of Pup-GGQ was observed after 4 h when using either the WT or Opengate CP (Figure **5A**). This *in vitro* result is consistent with in-cell observations of the proteasome complex reconstituted in *E. coli*.

To determine if the absence of Pup-GGQ degradation is a result of using *Msm* Mpa, purified *Mtb* Mpa was substituted for *Msm* Mpa in the degradation reaction [15]. No degradation of Pup-GGQ was observed when using either the Opengate (Figure **S6A**) or WT CP in combination with *Mtb* Mpa (Figure **S6B**). We concluded that Pup-GGQ is not a substrate for the mycobacterial proteasome CP.

To verify that the purified *Mtb* proteasome CPs are active, a known substrate, *N*-Succinyl-Leu-Leu-Val-Tyr-7-amino-4-methylcoumarin, Suc-LLVY-7-AMC, was used in the activity assay; when cleaved the AMC moiety fluoresces [13]. Suc-LLVY-7-AMC was added to purified WT or Opengate CP, and, over time, an increase in fluorescence was observed, consistent with proteasome-induced degradation of the substrate (Figure **5B**). To confirm that the increase in fluorescence is due to cleavage and not from contaminating proteolytic activity, a specific proteasome inhibitor, Bortezomib [35], which forms a tetrahedral adduct with the catalytic T10 in the PrcB subunit [14], was added to increasing concentrations of WT proteasome CP. Over time, in the presence of Bortezomib, fluorescence emission was reduced verifying that substrate degradation is due specifically to *Mtb* proteasome activity (Figure **5C**).

## Discussion

Assembly of macromolecular machinery in the presence of native ligands in the crowded cytosol presents a complicated system for study by amino-acid residue resolution techniques. We used in-cell STINT NMR to map the interactions of the prokaryotic ubiquitin-like protein, Pup, with the mycobacterial proteasome in *E. coli*. The intracellular medium provides a prokaryotic environment for structural study of *Mtb* proteasome function without the complications of additional factors that may specifically interact with this system [31]. Reconstructing the interactions between the mycobacterial *Msm* Mpa/*Mtb* proteasome CP complex and Pup-GGQ inside a cell at amino-acid residue resolution has allowed us to examine intracellular processes that are not accessible by *in vitro* investigations.

*In vitro* studies showed that Pup is a disordered protein possessing a transient helical structure in its C terminal region [22,23]. As in the case of α-synuclein, physiological conditions result in a seemingly disordered protein [30] may acquire stable secondary and even tertiary structure [36,37]. Only minor changes in the in-cell NMR spectrum of Pup-GGQ occur when compared to the cell lysate spectrum. This suggests that Pup-GGQ does not possess a stable secondary structure in the cytosol. Since Pup-GGQ acts as an anchor for the proteasome system, with the N-terminus assuming an extended structure [21,38], the disorder may be important for its function.

In-cell, Pup-GGQ interacts with the hexameric proteasomal ATPase, *Msm* Mpa, and its interaction surface does not change with the order of expression. This result was expected since intramolecular binding of Mpa subunits is very tight; the complex fails to dissociate on a chromatographic sizing column [10], consistent with an association constant greater than ~10^8^ M^-1^ [39]. Pup-GGQ binds to *Msm* Mpa primarily via the C-terminal half of the helical region. In addition, a short region of the N-terminus, T6-R8, and C-terminus, E52-Q64, are affected by *Msm* Mpa binding. Titrating *Msm* Mpa into [*U-* ^15^N] Pup-GGQ *in vitro* results in a gradual uniform broadening of the peaks from residues S21 to Q60 [23], excluding the C-terminal residues, K61, G62, G63 and Q64. In-cell, peak intensities do not decrease for all the residues in the Pup-GGQ helix, S21-A51. We suggest that this difference is due to the interactions of Pup-GGQ with components of cytosol that block part of the interaction surface between Pup and Mpa observed *in vitro*. The in-cell observations are also in general agreement with specific contacts in the helical region, identified in the Pup-Mpa co-crystal [21], although in this instance, truncated Mpa was used. The use of a truncated Mpa may not accurately represent the Pup-Mpa interaction due to the possibility of altered conformations resulting from the truncation and from *in vitro* conditions that fail to duplicate the cellular environment in which this interaction normally occurs.

In cells expressing Pup-GGQ, *Msm* Mpa, and WT or Opengate proteasome CPs, the intracellular concentration of CP is significantly less than that of *Msm* Mpa and Pup-GGQ (Figure **4D and 4E**). In this case, the Pup-GGQ/*Msm* Mpa complex will be the predominant species (Figures **3** and **4**). Nevertheless, the in-cell NMR spectrum of the Pup-GGQ/*Msm* Mpa complex in cells expressing non-stoichiometric amounts of proteasome CPs is different from that of cells expressing only Pup-GGQ and *Msm* Mpa: the presence of proteasome CPs results in the complete broadening of peaks associated with the N-terminal tail of Pup-GGQ. The Pup-GGQ/*Msm* Mpa complex appears to be stabilized by non-stoichiometric amounts of proteasome CPs. Since the proteasome CP binds to *Msm* Mpa with low affinity [14], we postulate that transient binding of the proteasome CP to the Pup-GGQ/*Msm* Mpa complex results in the N-terminal tail of Pup-GGQ being occluded by the *Msm* Mpa central cavity, which leads to complete broadening of the Pup-GGQ spectrum (Figure **3C**). Different in-cell spectra result when the order of over-expression of [*U-* ^15^N] Pup-GGQ and the *Msm* Mpa/proteasome CP complex are reversed (Figures **3** and **4**). The *Msm* Mpa/proteasome CP complex can take up to 12 hours to fully assemble in the cell following induction of over-expression. When [*U-* ^15^N] Pup-GGQ is over-expressed first, the result is a mixed population of cells containing free Pup-GGQ, Pup-GGQ bound to *Msm* Mpa and Pup-GGQ bound to the *Msm* Mpa/proteasome complex. The corresponding in-cell NMR spectrum will represent an average of these three spectra. In this case, the spectrum is very similar to that of [*U-* ^15^N] Pup-GGQ in complex with *Msm* Mpa, reflecting the interaction between *Msm* Mpa and the helical region of Pup-GGQ (Figure **2**). When the *Msm* Mpa/proteasome CP complex is over-expressed first, the in-cell spectrum is completely broadened. Western analysis demonstrated that this is not due to degradation of Pup-GGQ (Figure **4**).

**Figure 6:**
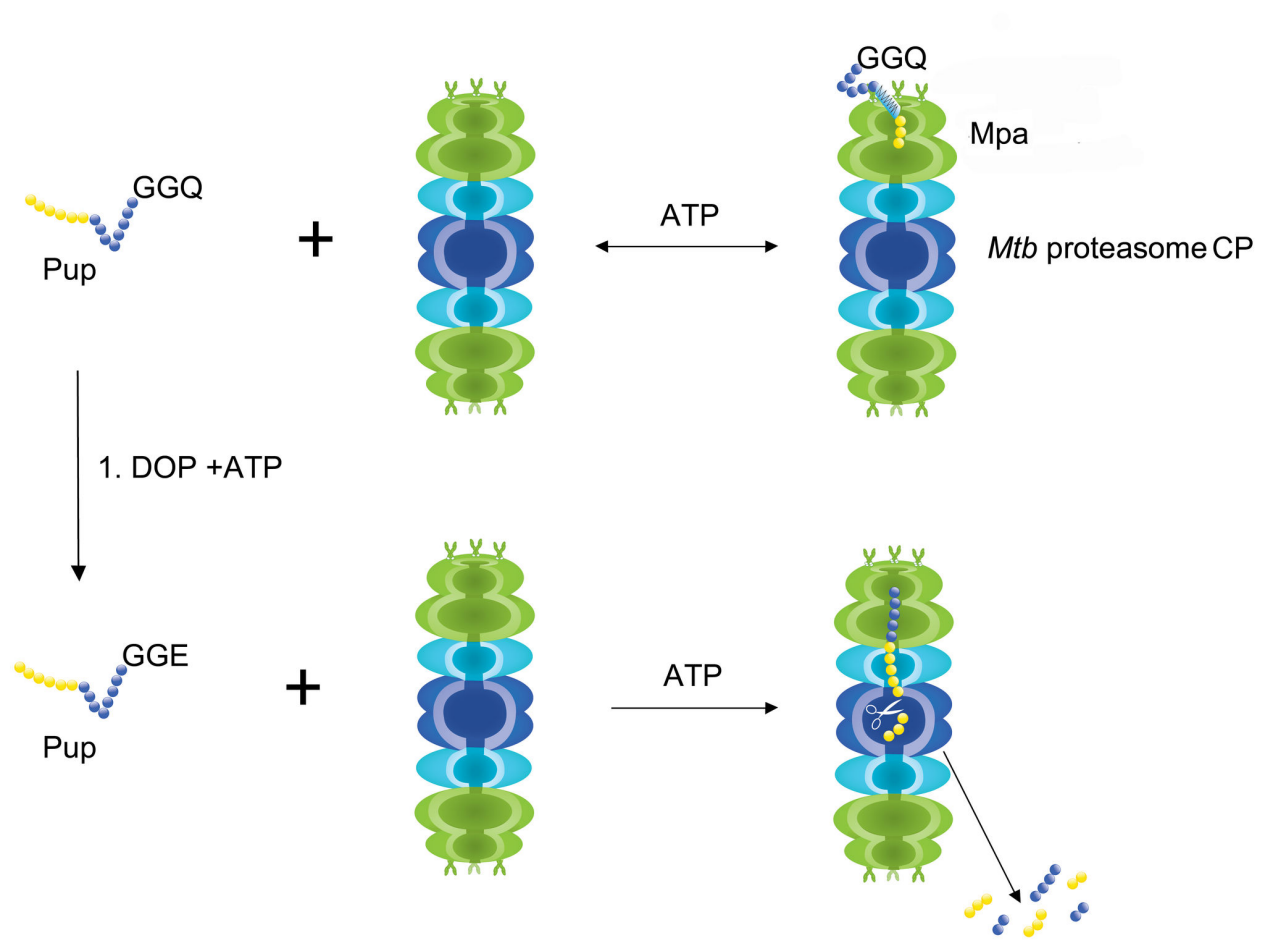
The precursor Pup-GGQ must be deamidated to Pup-GGE to activate proteasome-mediated degradation. Pup-GGQ is reversibly bound to the Mpa-proteasome CP complex, with the C terminus (blue) binding to the mouth of the hexamer and the N-terminus (yellow) falling into the central cavity, and is not degraded. Upon deamidation by Deamidase of Prokaryotic Ubiquitin like Protein (DOP) in the presence of ATP, Pup-GGE is irreversibly bound to the Mpa-proteasome CP complex and possibly degraded.

Unlike Ubiquitin, which is recycled in the eukaryotic proteasome [17], both free and target-bound Pup-GGE are degraded in the mycobacterium proteasome [15], but may be recycled by depupylase/deamidase Dop [34,40]. Pup-GGQ is a precursor molecule that is converted to Pup-GGE before being ligated to a substrate targeted for degradation [26,41]. Unlike Pup-GGE, which is a very poor substrate [15], Pup-GGQ is a not a substrate for proteasomal degradation, consistent with its function as a precursor molecule. Indeed, free Pup-GGQ can be detected in Dop mutants of both *M. smegmatis* and *M. tuberculosis* strains [33,42], albeit in low concentration. STINT-NMR experiments present a dynamic picture of the fate of Pup-GGQ inside a cell containing Mpa and proteasome CPs (Figure **6**): In the absence of the proteasome CP, the C- and N-terminal regions of Pup-GGQ interact with *Msm* Mpa only weakly; non-specific interactions are blocked by the cytosol. In the presence of non-stoichiometric amounts of proteasome CP, Pup-GGQ is completely bound to *Msm* Mpa with the C terminus binding to the mouth of the hexamer and the N-terminus falling into the central cavity (Figures **3** and **6**). The charge difference between glutamine and glutamate in Pup may affect proteasomal degradation.

In conclusion, we assembled a functional 1.2 megadalton mycobacterium proteasome, consisting of Mpa and the proteasome CP, inside *E. coli* to study its interactions with the prokaryotic ubiquitin-like protein, Pup-GGQ at amino acid residue resolution. By using STINT-NMR, we show that in-cell proteasomal degradation is dynamically regulated by transient interactions between Mpa and the proteasome CP. Differences between the binding of Pup and Mpa in vivo and *in vitro* underscore the importance of studying interactions under close to physiological conditions.

## Supporting Information

Figure S1 **Pup-GGQ is disordered inside the cell.** A. Cell leakage test. An in-cell NMR sample of overexpressed [*U*-^15^N] Pup-GGQ was re-suspended in 500 µL of NMR buffer, 10 mM KPO_4_, pH 7.0, and incubated for 1 hour at RT. The cells were pelleted and the ^1^H{^15^N}-HSQC spectrum of the supernatant was collected. No NMR signal was observed above the noise level implying that no leakage or cell lysis was occurring during the experimental acquisition time. B. Differences in the chemical shifts of ^15^N-HSQC spectra between in-cell and *in vitro* Pup-GGQ. The changes in chemical shifts of amide nitrogens and covalently attached amide protons, Δ, were calculated by using Δ = (δ_H_^2^ + (δ_N_/4)^2^)^^1/2^^, where δ_H(N)_ represents the change in hydrogen and nitrogen chemical shifts. Based on the comparison of chemical shifts, we conclude that in-cell Pup-GGQ does not contain any structure induced by macromolecular crowding. C. Differences in peak intensities of the ^15^N-HSQC spectra between in-cell and *in vitro* Pup-GGQ. Since the ^1^H{^15^N}-HSQC peaks of side chain amide protons and nitrogens of [*U*-^15^N] Pup-GGQ glutamines do not change their positions in in-cell and *in vitro* Pup-GGQ, we used the intensities of these peaks (I_ref_) to scale the intensities of backbone amide protons and nitrogens. Changes in intensity were calculated by using ΔI = ((I/I_ref_)_*in_vitro*_ – (I/I_ref_)_in-cell_)/(I/I_ref_)_*in_vitro*_, where (I/I_ref_)_in-cell_ is the scaled intensity of an individual peak in the in-cell spectrum of Pup-GGQ and (I/I_ref_)_*in_vitro*_ is the scaled intensity of individual peaks in the *in vitro* spectrum of Pup-GGQ. Positive changes in intensity reflect peak broadening due to the decrease in viscosity of the lysate relative to that of the cytosol. Changes in the chemical shifts or intensities above the continuous lines are considered to be significant.
(PDF)

Figure S2 **Pup-GGQ forms multiple contacts with *Msm* Mpa.** In **A**. and **B**, *Msm* Mpa was over-expressed first followed by over-expression of [*U*- ^15^N] Pup-GGQ. **A**. Differences in the chemical shifts of the ^15^N-HSQC spectra between free Pup-GGQ and the Pup-GGQ/ *Msm* Mpa complex. **B**. Relative changes in peak intensities of the ^15^N-HSQC spectra between free Pup-GGQ and the Pup-GGQ/ *Msm* Mpa complex. In **C**. and **D**, [*U*- ^15^N] Pup-GGQ was over-expressed first followed by over-expression of *Msm* Mpa. **C**. Differences in the chemical shifts of the ^15^N-HSQC spectra between free Pup-GGQ and Pup-GGQ in complex with *Msm* Mpa. D. Relative changes in peak intensities of the ^15^N-HSQC spectra between free Pup-GGQ and Pup-GGQ in complex with *Msm* Mpa. The order of over-expression of *Msm* Mpa and Pup-GGQ does not change the Pup-GGQ-*Msm* Mpa interaction. The changes in chemical shifts of amide nitrogens and covalently attached amide protons Δ(ppm) and changes in peaks intensities ΔI were calculated as described in Materials and Methods. Overlapped peaks in the Pup-GGQ-Mpa complex are indicated by crosses. Changes in the chemical shifts or intensities above the continuous lines are considered to be significant.
(PDF)

Figure S3 **Pup-GGQ is extensively engaged by the *Msm* Mpa/WT proteasome CP complex in-cell.** In **A**. and **B**, *Msm* Mpa and the WT proteasome CP were simultaneously over-expressed first followed by over-expression of [*U*- ^15^N] Pup-GGQ. **B**. Differences in the chemical shifts of the ^15^N-HSQC spectra between free Pup-GGQ and the Pup-GGQ-*Msm* Mpa/WT proteasome CP complex. **C**. Relative changes in peak intensities of the ^15^N-HSQC spectra between free Pup-GGQ and the Pup-GGQ/ *Msm* Mpa/WT proteasome CP complex. In **D**. and **E**, [*U*- ^15^N] Pup-GGQ was over-expressed first followed by simultaneous over-expression of *Msm* Mpa and the WT proteasome CP. In this case, the *Msm* Mpa/Opengate proteasome CP complex interacts with the C-terminus of Pup-GGQ. **D**. Differences in the chemical shifts of the ^15^N-HSQC spectra between free Pup-GGQ and Pup-GGQ in complex with the *Msm* Mpa/WT proteasome CP complex. **E**. Relative changes in peak intensities of the ^15^N-HSQC spectra between free Pup-GGQ and Pup-GGQ in complex with the *Msm* Mpa/WT proteasome CP complex. The changes in chemical shifts of amide nitrogens and covalently attached amide protons Δ(ppm) and changes in peaks intensities ΔI were calculated as described in Materials and Methods. Overlapped peaks in the Pup-GGQ- *Msm* Mpa/WT proteasome CP complex are indicated by crosses. Filled dots above the bars indicate that the peaks are at the noise level. Changes in the chemical shifts or intensities above the continuous lines are considered to be significant.
(PDF)

Figure S4 **Pup-GGQ is extensively engaged by the *Msm* Mpa/Opengate proteasome CP complex in-cell.** In **A**. and **B**. *Msm* Mpa and the Opengate proteasome CP were over-expressed first followed by over-expression of [*U*- ^15^N] Pup-GGQ. **A**. Differences in the chemical shifts of the ^15^N-HSQC spectra between free Pup-GGQ and Pup-GGQ in complex with the *Msm* Mpa/Opengate proteasome CP complex. **B**. Relative changes in peak intensities of the ^15^N-HSQC spectra between free Pup-GGQ and the Pup-GGQ/*Msm* Mpa/Opengate proteasome CP complex. In **C**. and **D**, [*U*- ^15^N] Pup-GGQ was over-expressed first followed by over-expression of *Msm* Mpa and the Opengate proteasome CP. In this case, the *Msm* Mpa/Opengate proteasome CP complex interacts with the C-terminus of Pup-GGQ. **C**. Differences in the chemical shifts of the ^15^N-HSQC spectra between free Pup-GGQ and Pup-GGQ in complex with the *Msm* Mpa/Opengate proteasome CP complex. **D**. Relative changes in peak intensities of the ^15^N-HSQC spectra between free Pup-GGQ and Pup-GGQ in complex with the *Msm* Mpa/Opengate proteasome CP complex. The changes in chemical shifts of amide nitrogens and covalently attached amide protons Δ(ppm) and changes in peaks intensities ΔI were calculated as described in Materials and Methods. Overlapped peaks in the *Msm* Mpa/Opengate proteasome CP complex are indicated by crosses. Filled dots above the bars indicate that the peaks are at the noise level. Changes in the chemical shifts or intensities above the continuous lines are considered to be significant.
(PDF)

Figure S5 **Over-expression of *Msm* Mpa and the Opengate proteasome CP suppresses Pup-GGE over-expression.** (top panel) Western blot showing over-expression of Pup-GGE alone or following simultaneous over-expression of *Msm* Mpa and the Opengate proteasome CP. Samples were collected 6, 12 and 24 h post-Pup-GGE induction. Material loaded into each lane was normalized by the OD of the cell culture (lower panel). Intracellular Pup-GGE decreases over time in the presence of *Msm* Mpa and the Opengate proteasome CP. Pup-GGE bands were quantified using a Bio-Rad ChemiDoc XRS imager. The integrated density per mm^2^ (Density INT/mm^2^) is shown. The experiments were repeated in triplicate.
(TIF)

Figure S6 **Pup-GGQ is not degraded in the presence of the *Mtb* Mpa/WT or Opengate proteasome complex *in vitro*.** **A**. SDS-PAGE of the in vitro degradation assay of Pup-GGQ by the *Mtb* Mpa/Opengate proteasome CP complex. PrcB and PrcAΔ7N are the Opengate proteasome CP β- and α-subunits, respectively. **B**. SDS-PAGE of the in vitro degradation assay of Pup-GGQ by the *Mtb* Mpa/WT proteasome CP complex. PrcB and PrcA are the WT proteasome CP β- and α-subunits, respectively.
(TIF)
