# Supplementary figures and images for: Fate of Pup inside the Mycobacterium Proteasome Studied by in-Cell NMR
Source: PLoS One. 2013 Sep 10;8(9):e74576. doi: 10.1371/journal.pone.0074576 (PMC3769308; doi:10.1371/journal.pone.0074576)

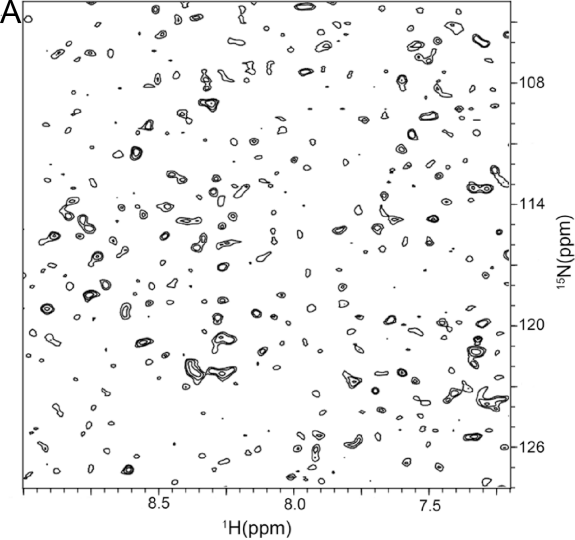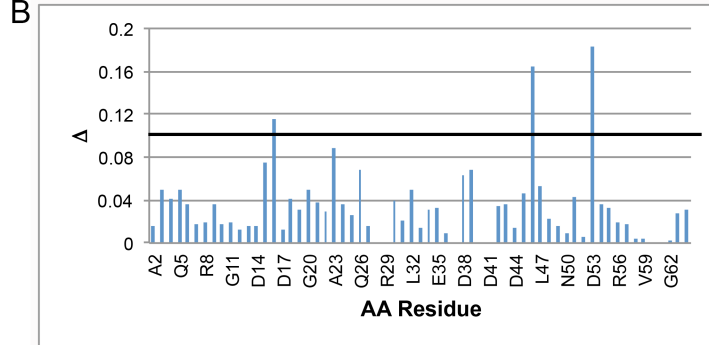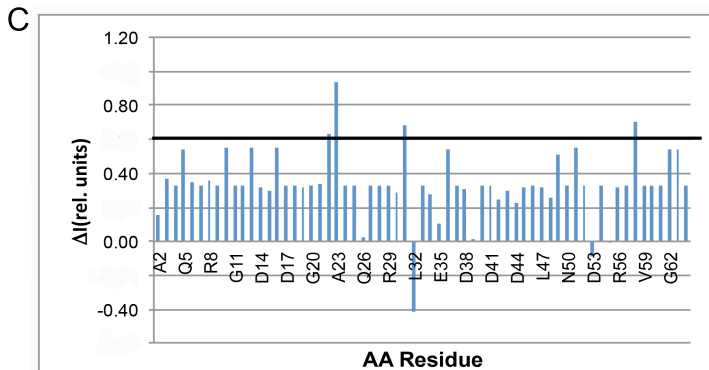

Supplement: Figure S1 — Pup-GGQ is disordered inside the cell. A. Cell leakage test. An in-cell NMR sample of overexpressed [U-15N] Pup-GGQ was re-suspended in 500 µL of NMR buffer, 10 mM KPO4, pH 7.0, and incubated for 1 hour at RT. The cells were pelleted and the 1H{15N}-HSQC spectrum of the supernatant was collected. No NMR signal was observed above the noise level implying that no leakage or cell lysis was occurring during the experimental acquisition time. B. Differences in the chemical shifts of 15N-HSQC spectra between in-cell and in vitro Pup-GGQ. The changes in chemical shifts of amide nitrogens and covalently attached amide protons, Δ, were calculated by using Δ = (δH 2 + (δN/4)2)1/2, where δH(N) represents the change in hydrogen and nitrogen chemical shifts. Based on the comparison of chemical shifts, we conclude that in-cell Pup-GGQ does not contain any structure induced by macromolecular crowding. C. Differences in peak intensities of the 15N-HSQC spectra between in-cell and in vitro Pup-GGQ. Since the 1H{15N}-HSQC peaks of side chain amide protons and nitrogens of [U-15N] Pup-GGQ glutamines do not change their positions in in-cell and in vitro Pup-GGQ, we used the intensities of these peaks (Iref) to scale the intensities of backbone amide protons and nitrogens. Changes in intensity were calculated by using ΔI = ((I/Iref)in_vitro – (I/Iref)in-cell)/(I/Iref)in_vitro, where (I/Iref)in-cell is the scaled intensity of an individual peak in the in-cell spectrum of Pup-GGQ and (I/Iref)in_vitro is the scaled intensity of individual peaks in the in vitro spectrum of Pup-GGQ. Positive changes in intensity reflect peak broadening due to the decrease in viscosity of the lysate relative to that of the cytosol. Changes in the chemical shifts or intensities above the continuous lines are considered to be significant. (PDF) [file pone.0074576.s001.pdf]

A

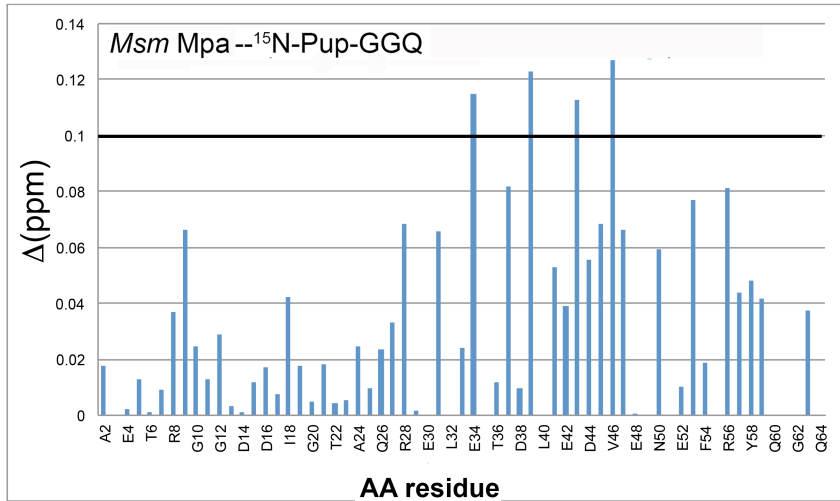

B

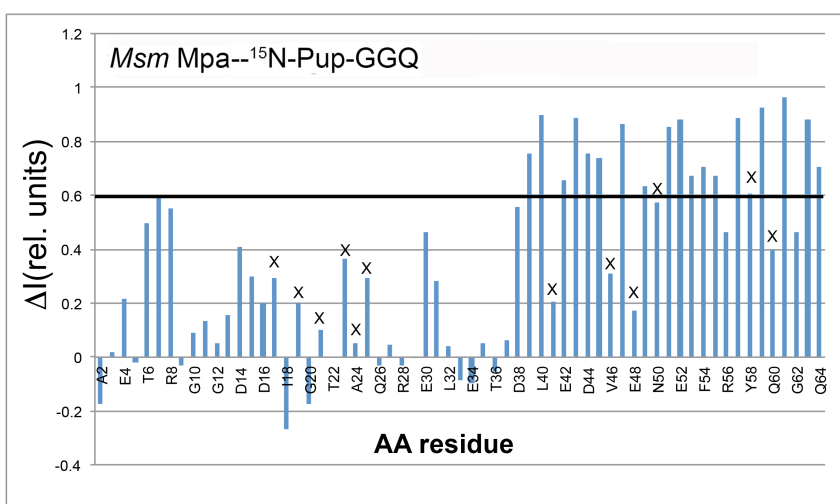

C

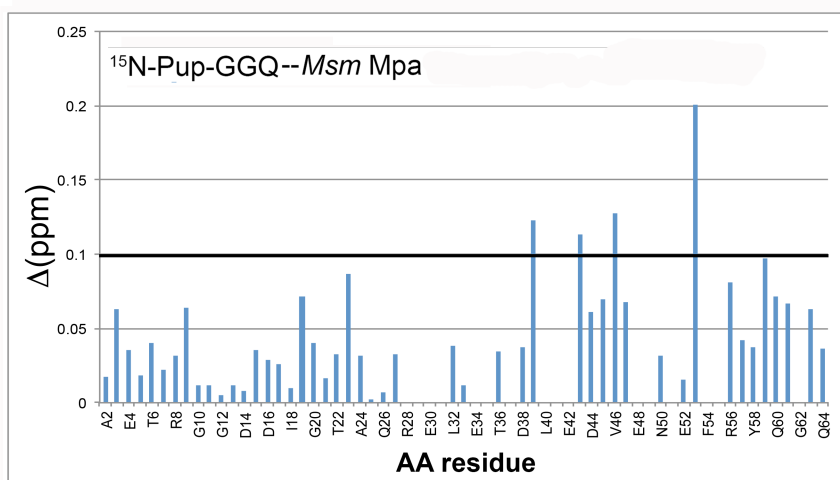

D

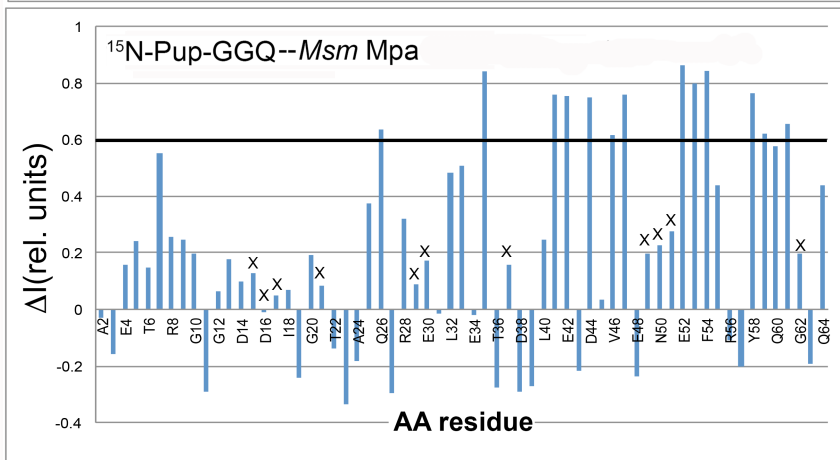

Supplement: Figure S2 — Pup-GGQ forms multiple contacts with Msm Mpa. In A. and B, Msm Mpa was over-expressed first followed by over-expression of [U- 15N] Pup-GGQ. A. Differences in the chemical shifts of the 15N-HSQC spectra between free Pup-GGQ and the Pup-GGQ/ Msm Mpa complex. B. Relative changes in peak intensities of the 15N-HSQC spectra between free Pup-GGQ and the Pup-GGQ/ Msm Mpa complex. In C. and D, [U- 15N] Pup-GGQ was over-expressed first followed by over-expression of Msm Mpa. C. Differences in the chemical shifts of the 15N-HSQC spectra between free Pup-GGQ and Pup-GGQ in complex with Msm Mpa. D. Relative changes in peak intensities of the 15N-HSQC spectra between free Pup-GGQ and Pup-GGQ in complex with Msm Mpa. The order of over-expression of Msm Mpa and Pup-GGQ does not change the Pup-GGQ-Msm Mpa interaction. The changes in chemical shifts of amide nitrogens and covalently attached amide protons Δ(ppm) and changes in peaks intensities ΔI were calculated as described in Materials and Methods. Overlapped peaks in the Pup-GGQ-Mpa complex are indicated by crosses. Changes in the chemical shifts or intensities above the continuous lines are considered to be significant. (PDF) [file pone.0074576.s002.pdf]

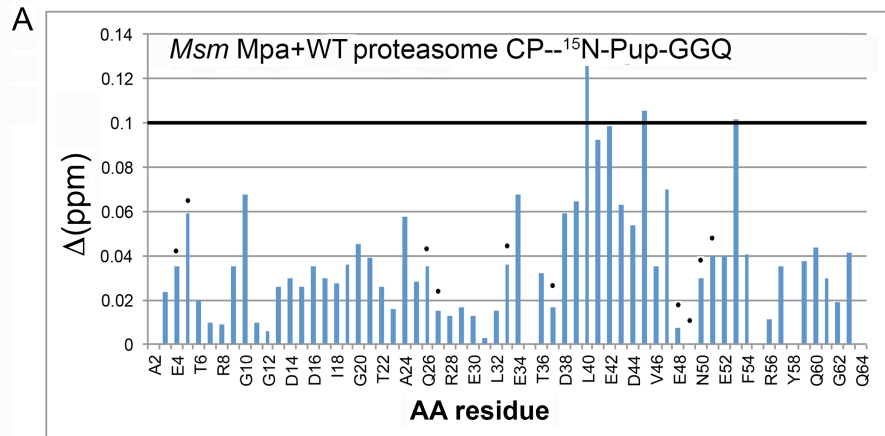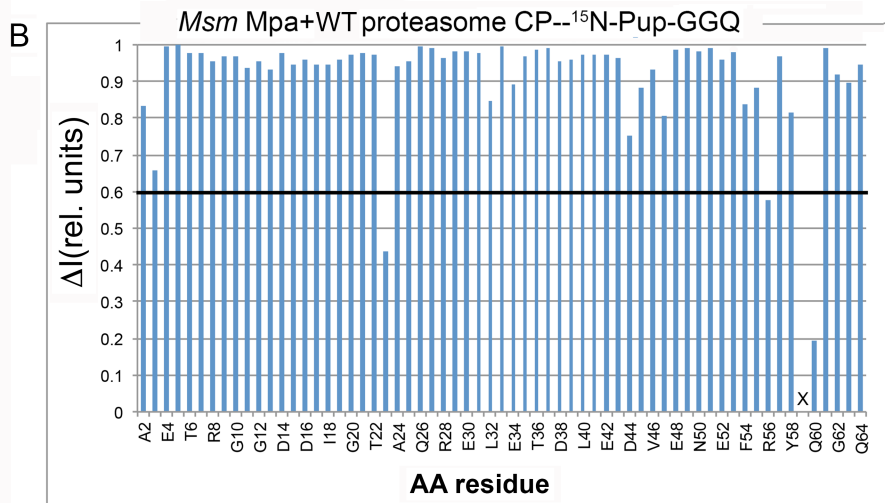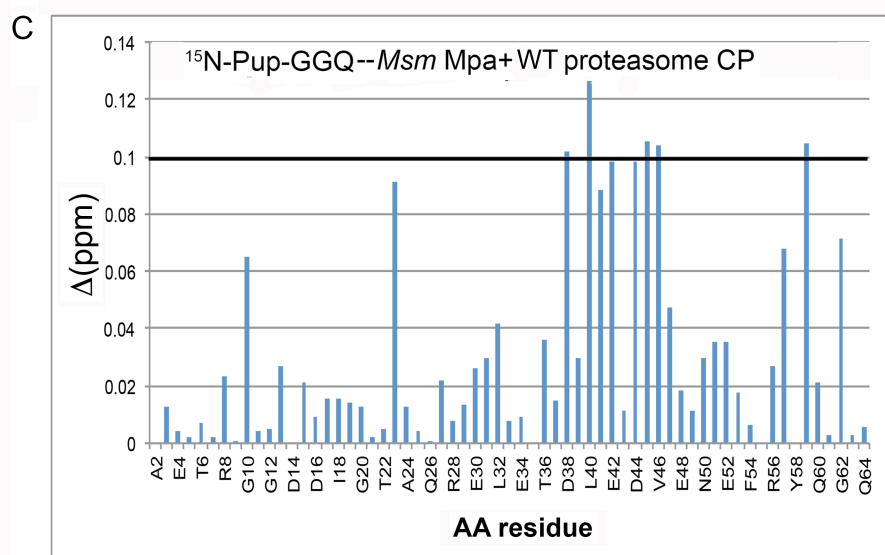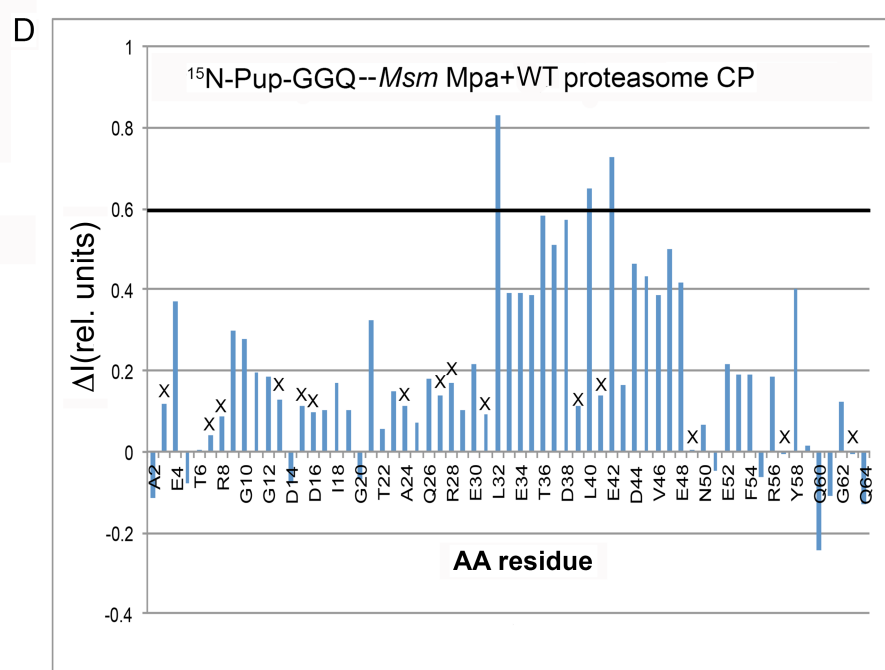

Supplement: Figure S3 — Pup-GGQ is extensively engaged by the Msm Mpa/WT proteasome CP complex in-cell. In A. and B, Msm Mpa and the WT proteasome CP were simultaneously over-expressed first followed by over-expression of [U- 15N] Pup-GGQ. B. Differences in the chemical shifts of the 15N-HSQC spectra between free Pup-GGQ and the Pup-GGQ-Msm Mpa/WT proteasome CP complex. C. Relative changes in peak intensities of the 15N-HSQC spectra between free Pup-GGQ and the Pup-GGQ/ Msm Mpa/WT proteasome CP complex. In D. and E, [U- 15N] Pup-GGQ was over-expressed first followed by simultaneous over-expression of Msm Mpa and the WT proteasome CP. In this case, the Msm Mpa/Opengate proteasome CP complex interacts with the C-terminus of Pup-GGQ. D. Differences in the chemical shifts of the 15N-HSQC spectra between free Pup-GGQ and Pup-GGQ in complex with the Msm Mpa/WT proteasome CP complex. E. Relative changes in peak intensities of the 15N-HSQC spectra between free Pup-GGQ and Pup-GGQ in complex with the Msm Mpa/WT proteasome CP complex. The changes in chemical shifts of amide nitrogens and covalently attached amide protons Δ(ppm) and changes in peaks intensities ΔI were calculated as described in Materials and Methods. Overlapped peaks in the Pup-GGQ- Msm Mpa/WT proteasome CP complex are indicated by crosses. Filled dots above the bars indicate that the peaks are at the noise level. Changes in the chemical shifts or intensities above the continuous lines are considered to be significant. (PDF) [file pone.0074576.s003.pdf]

A

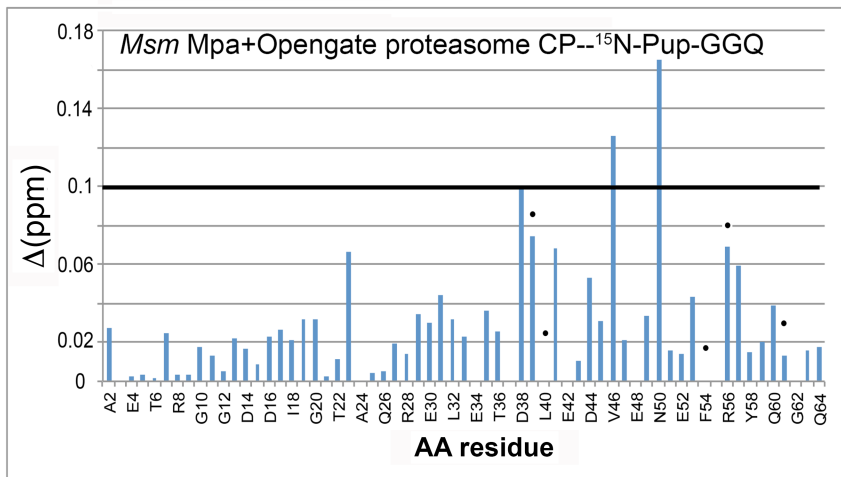

B

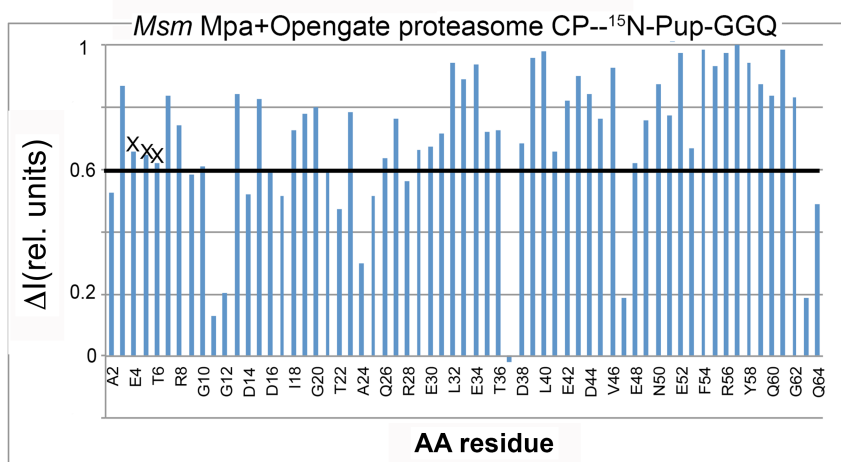

C

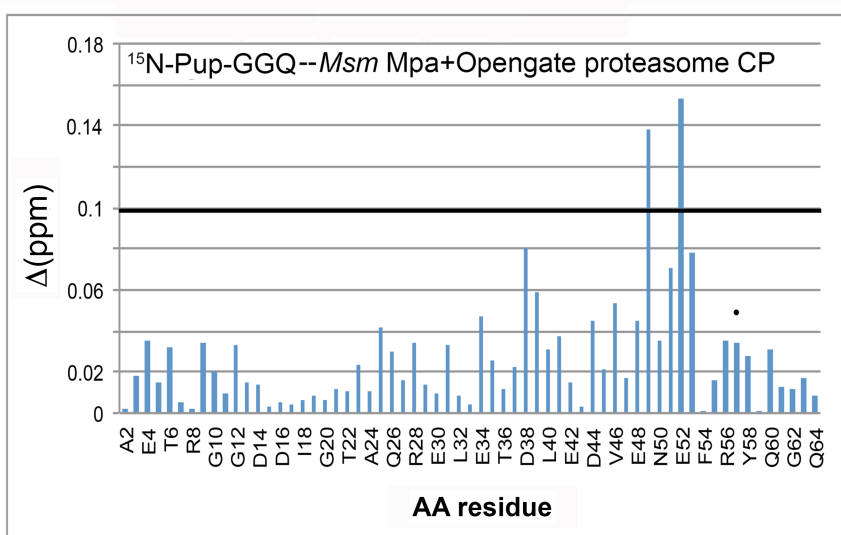

D

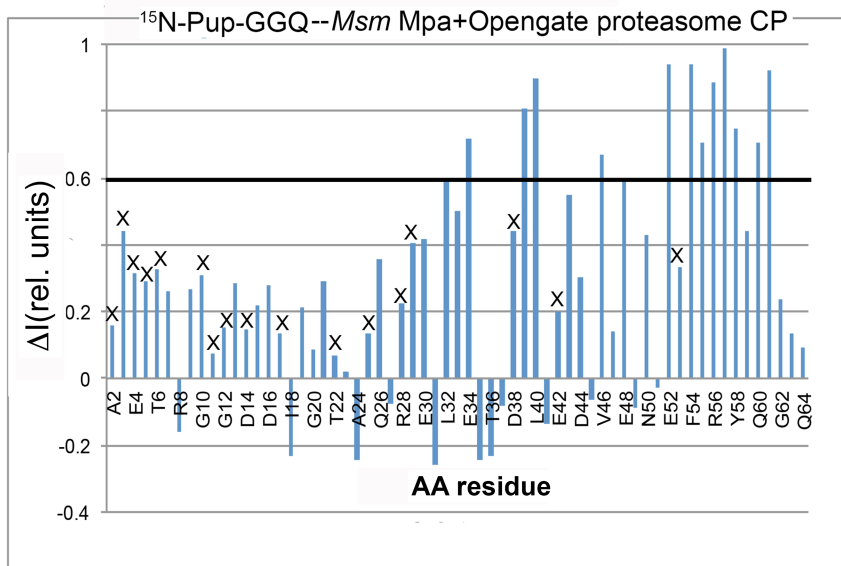

Supplement: Figure S4 — Pup-GGQ is extensively engaged by the Msm Mpa/Opengate proteasome CP complex in-cell. In A. and B. Msm Mpa and the Opengate proteasome CP were over-expressed first followed by over-expression of [U- 15N] Pup-GGQ. A. Differences in the chemical shifts of the 15N-HSQC spectra between free Pup-GGQ and Pup-GGQ in complex with the Msm Mpa/Opengate proteasome CP complex. B. Relative changes in peak intensities of the 15N-HSQC spectra between free Pup-GGQ and the Pup-GGQ/Msm Mpa/Opengate proteasome CP complex. In C. and D, [U- 15N] Pup-GGQ was over-expressed first followed by over-expression of Msm Mpa and the Opengate proteasome CP. In this case, the Msm Mpa/Opengate proteasome CP complex interacts with the C-terminus of Pup-GGQ. C. Differences in the chemical shifts of the 15N-HSQC spectra between free Pup-GGQ and Pup-GGQ in complex with the Msm Mpa/Opengate proteasome CP complex. D. Relative changes in peak intensities of the 15N-HSQC spectra between free Pup-GGQ and Pup-GGQ in complex with the Msm Mpa/Opengate proteasome CP complex. The changes in chemical shifts of amide nitrogens and covalently attached amide protons Δ(ppm) and changes in peaks intensities ΔI were calculated as described in Materials and Methods. Overlapped peaks in the Msm Mpa/Opengate proteasome CP complex are indicated by crosses. Filled dots above the bars indicate that the peaks are at the noise level. Changes in the chemical shifts or intensities above the continuous lines are considered to be significant. (PDF) [file pone.0074576.s004.pdf]

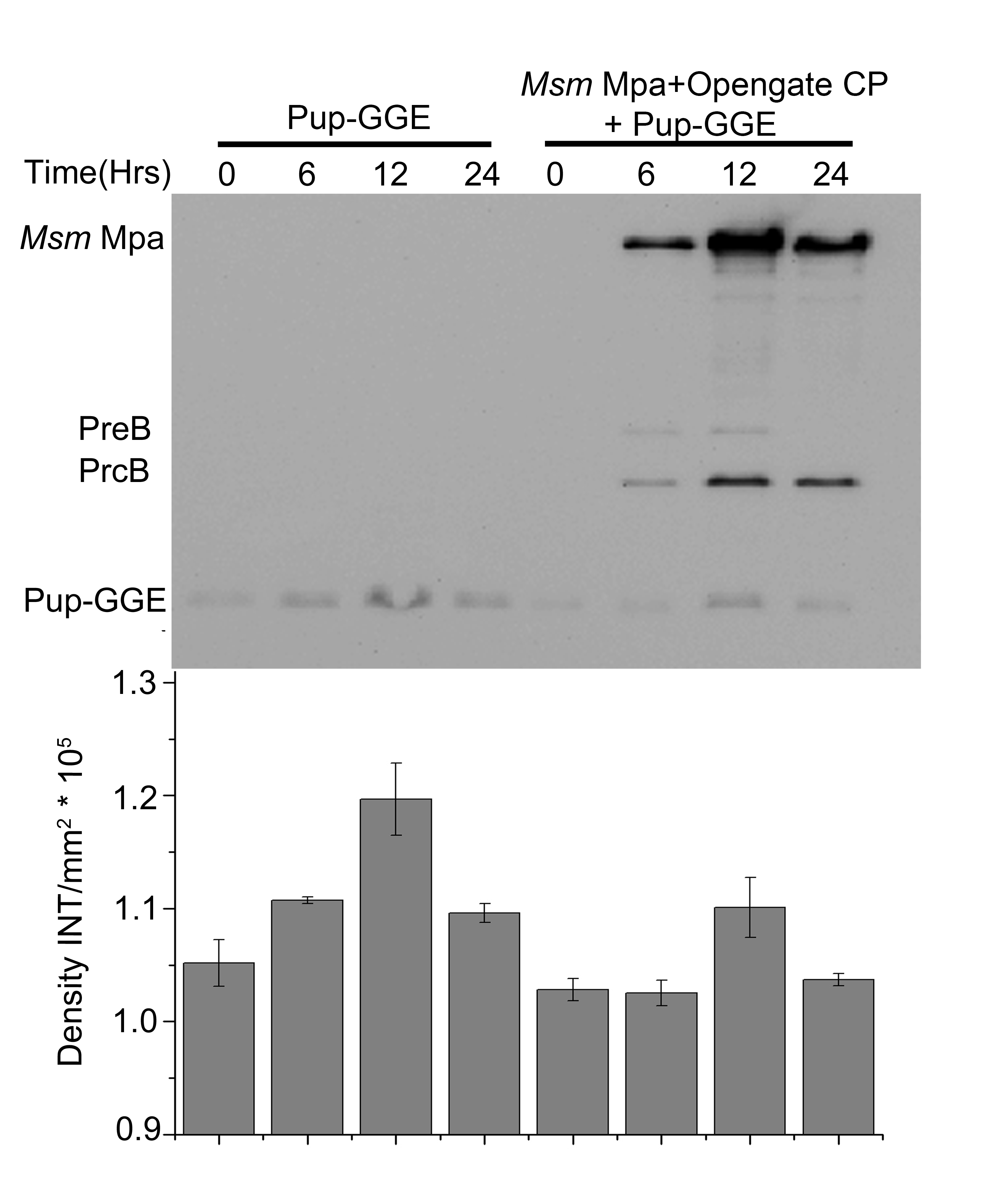

Supplement: Figure S5 — Over-expression of Msm Mpa and the Opengate proteasome CP suppresses Pup-GGE over-expression. (top panel) Western blot showing over-expression of Pup-GGE alone or following simultaneous over-expression of Msm Mpa and the Opengate proteasome CP. Samples were collected 6, 12 and 24 h post-Pup-GGE induction. Material loaded into each lane was normalized by the OD of the cell culture (lower panel). Intracellular Pup-GGE decreases over time in the presence of Msm Mpa and the Opengate proteasome CP. Pup-GGE bands were quantified using a Bio-Rad ChemiDoc XRS imager. The integrated density per mm2 (Density INT/mm2) is shown. The experiments were repeated in triplicate. (TIF) [file pone.0074576.s005.tif]

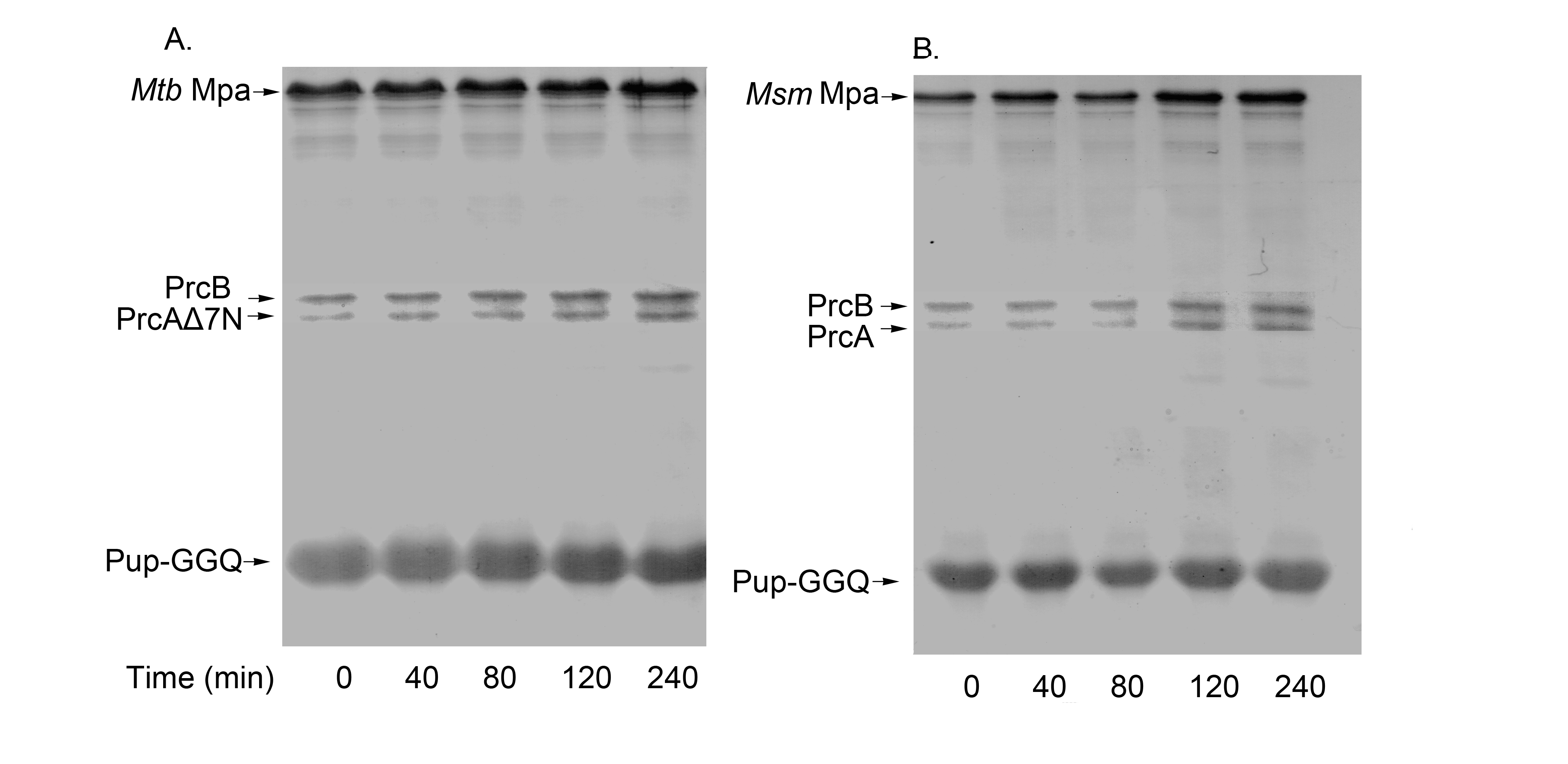

Supplement: Figure S6 — Pup-GGQ is not degraded in the presence of the Mtb Mpa/WT or Opengate proteasome complex in vitro. A. SDS-PAGE of the in vitro degradation assay of Pup-GGQ by the Mtb Mpa/Opengate proteasome CP complex. PrcB and PrcAΔ7N are the Opengate proteasome CP β- and α-subunits, respectively. B. SDS-PAGE of the in vitro degradation assay of Pup-GGQ by the Mtb Mpa/WT proteasome CP complex. PrcB and PrcA are the WT proteasome CP β- and α-subunits, respectively. (TIF) [file pone.0074576.s006.tif]
